# Blinded‐Into‐Unblinded Interim Analyses for Clinical Trials With Time‐to‐Event Endpoints

**DOI:** 10.1002/bimj.70151

**Published:** 2026-07-24

**Authors:** Stephen Schüürhuis, Jan Meis, Björn Bokelmann, Meinhard Kieser, Carolin Herrmann

**Affiliations:** ^1^ Institute of Biometry and Clinical Epidemiology, Charité ‐ Universitätsmedizin Berlin Corporate member of Freie Universität Berlin and Humboldt‐Universität zu Berlin Berlin Germany; ^2^ Institute of Medical Biometry University of Heidelberg Heidelberg Germany; ^3^ Mathematical Institute and Center for Digital Medicine Heinrich Heine University Düsseldorf Düsseldorf Germany

**Keywords:** adaptive design, group‐sequential design, sample size, time‐to‐event analysis

## Abstract

A large proportion of clinical trials do not meet their recruitment targets. Trials using time‐to‐event endpoints come along with the additional complexity that the amount of available information depends on the number of observed events, which is random for a specified point in time. This means that even if a trial may have recruited the planned number of patients in its recruitment interval, the amount of available information at a fixed calendar time is random.

The determination of the sample size underlying a clinical trial is a very important aspect when designing a trial. When adaptations of the pre‐specified sample size are a desired design option to address the uncertainty of underlying assumptions, blinded interim analyses are recommended by the authorities. However, blinding cannot be assured when there is a high interest in a potential early trial stop or a high uncertainty about the underlying effect size. In those situations, (adaptive) group‐sequential trial designs may be used. In this work, we propose a flexible, hybrid approach. Based on the chances of an early trial stop, either a blinded or an unblinded interim analysis is conducted such that the advantages of the two commonly applied approaches are combined. We focus on trials where the time to an event is the primary outcome. These studies usually come along with a long study duration, where the necessity of design adaptations may be especially appealing.

## 1 Introduction

The calculation of the sample size is one crucial aspect when designing clinical trials. Neither too large nor too small sample sizes are desirable both from the ethical but also from an economic perspective. If the sample size is too small, a potentially existent treatment effect might not be detected, hence resulting in spending patients and money on a project where no clear results are expected. On the other hand, if the sample size is too large, more patients than necessary are exposed to study‐related risks, and the trial comes along with higher costs. At the same time, one usually faces some uncertainty about the effect size and the value of nuisance parameters when calculating the sample size of a clinical trial. Hence, different solutions were developed to address this uncertainty. One solution strategy is group‐sequential designs that allow for an early stopping of the trial in case the effect at interim is much larger or smaller than anticipated and otherwise a continuation of the trial with the recruitment of further patients (cf., e.g., Jennison and Turnbull (1999) for an overview). Another option is adaptive group‐sequential trial designs, where design features of the trial can be modified, for example, an update of the sample size based on the observed interim effect size.

Trials in which the primary endpoint is a time‐to‐event endpoint often come with the additional challenge of a long trial duration. Here, it can be especially beneficial to have the option of modifying design aspects during the ongoing trial. At the same time, time‐to‐event endpoints are especially challenging in adaptive trial designs as every interim analysis, and also early terminations, need to deal with the pipeline data. To get an idea of the versatility of the approaches, we refer to a selection here. Whitehead et al. (2001) explain how blinded mid‐trial sample size adjustments can be made by observing the pooled survival function and comparing it to the anticipated survival function. The difference in survival functions can then be used to calculate the required number of subjects to reach the originally planned number of events. Schäfer and Müller (2001) describe how the conditional rejection probability approach can be applied in adaptive trials with time‐to‐event endpoints. Cook (2003) presents a Markov‐model‐based approach for blinded and unblinded design adaptations. Shen and Cai (2003) consider an adaptive multi‐stage design with recalculation of the number of required events and futility stops, though without early efficacy stops. The number of stages of the design is also adaptively updated. Li et al. (2005) consider an adaptive two‐stage design with a futility and efficacy stop and a recalculation based on the observed test‐statistic. Testing is done based on an inverse‐normal combination test with weights according to the planned information rates, and event number recalculation is done based on conditional power considerations. Wassmer (2006) shows how the inverse‐normal combination test can be used for analyzing multi‐stage group‐sequential survival trials and describes how to obtain point estimates, adjusted confidence intervals, and *p*‐values. Desseaux and Porcher (2007) go with the same approach as Li et al. (2005), but with Fisher's product combination test instead of the inverse‐normal combination test. Jahn‐Eimermacher and Ingel (2009) compare the use of log‐rank tests based on left‐truncation against the independent‐increments approach in the inverse normal combination method. Feld et al. (2021) present a sample size recalculation approach, which incorporates information on the estimated event rate as well as the log‐rank statistic to arrive at an interim decision for early stopping or sample size recalculation. Todd et al. (2012) apply the idea of using an unblinded interim analysis to estimate the event rate to trials with time‐to‐event endpoints. Adaptive trial designs for time‐to‐event endpoints are also applied in real trials. However, their reporting is limited (cf., e.g., Hade et al. 2019; Huang et al. 2018). For example, Hade et al. (2019) use blinded recalculation in a trial with a time‐to‐event outcome and report that it worked well. This is a follow‐up publication to their publication (Hade et al. 2010), where they describe the methodology of the recalculation. McClure et al. (2012) do a sample size adjustment in a real trial. Friede et al. (2019) suggest a blinded sample size reestimation approach that is related to a pharmaceutical clinical trial. Furthermore, the article by Mauer et al. (2012) needs to be referred to when it comes to application, as they discuss regulatory issues around adaptive designs in trials for time‐to‐event endpoints.

To summarize, there exists a broad range of methodological possibilities. At the same time, it needs to be decided whether the interim analysis is blinded or unblinded. Agencies tend to prefer blinded interim analyses when it comes to (planned) mid‐trial adaptations and require that the type‐1 error rate is not inflated (European Medicines Agency 2007; Food and Drug Administration 2018). However, depending on the reason for an interim analysis, the decision for an unblinded interim analysis might be made.

In this work, we present a hybrid approach. In this “blinded‐into‐unblinded” interim analysis, the observed pooled number of events at interim determines whether the interim analysis happens blinded or unblinded. Hence, unblinding is only pursued if it is deemed reasonable, and otherwise blinding is preserved as preferred by the agencies. To limit the scope of the paper, we only consider classical group‐sequential trial designs with this new adaptive feature (i.e., the decision on a blinded or unblinded interim analysis), but we do not evaluate the various alternative options of adaptive sample size recalculation in this work.

The paper is organized as follows: In a first step, we describe the underlying setting before introducing the hybrid approach in its three variants. After that, we present the results of an extensive simulation study as well as a short example for the application of the method to a clinical trial. We close with a discussion of the findings, further thoughts for application setting, and an outlook.

## 2 Methods

### 2.1 Setting and Hypothesis Test

We consider a randomized clinical trial with two groups, a treatment group A and a control group B, where the time to the occurrence of an event is of primary interest. More precisely, we assume proportional hazards and therefore a time‐independent hazard ratio θ, defined in terms of the two hazard rates $h_{A}\left(t\right)$ and $h_{B}\left(t\right)$, aiming to test the null hypothesis

$$\begin{array}{c} H_{0}:\theta :=\frac{h_{A}\left(t\right)}{h_{B}\left(t\right)}\geq 1\;\text{versus}\;H_{1}:\theta <1. \end{array}$$

Here, we assume that delaying the occurrence of an event represents a desirable effect of the new potential drug. For testing the stated one‐sided hypothesis, the well‐known log‐rank test, going back to Mantel et al. (1966) and Peto and Peto (1972), is used. For the sake of simplicity, we further assume that no ties in survival times are observed. Let $n_{i}^{\left(j\right)}$, j∈{A,B}, describe the number of patients at risk when the ith event occurs with i≤d, where d describes the total number of events that have occurred within the trial. Then, the log‐rank test statistic is given by

(1) $$\begin{array}{c} LR=\frac{\sum_{i=1}^{d}\left(d_{i}^{\left(B\right)}-\frac{n_{i}^{\left(B\right)}}{n_{i}^{\left(A\right)}+n_{i}^{\left(B\right)}}\right)}{\sqrt{\sum_{i=1}^{d}\frac{n_{i}^{\left(A\right)}\cdot n_{i}^{\left(B\right)}}{{\left(n_{i}^{\left(A\right)}+n_{i}^{\left(B\right)}\right)}^{2}}}}, \end{array}$$

which is known to asymptotically follow a standard normal distribution under $H_{0}$. Here, $d_{i}^{\left(B\right)}$ takes the value 1 if the ith event occurred in group B and it takes 0 if the ith event occurred in group A. In a trial design with an unblinded interim analysis, the log‐rank test statistic in (1) is calculated at both stages based on nested subsets of data. The resulting vector of cumulative test statistics ${\left(LR_{1},LR_{1+2}\right)}^{t}$ then asymptotically follows the bivariate normal distribution

(2) $$\begin{array}{c} \left(\begin{array}{c} LR_{1}\\ LR_{1+2} \end{array}\right)∼N_{2}\left(0,\left(\begin{array}{cc} 1 & \sqrt{I_{1}}\\ \sqrt{I_{1}} & 1 \end{array}\right)\right), \end{array}$$

under $H_{0}$ (see, e.g., Wassmer and Brannath 2016; Whitehead 1984), where $I_{1}$ denotes the information fraction available at interim.

### 2.2 Considered Trial Designs

As already pointed out in the introduction, the planning of a clinical trial usually comes along with insecurities about the underlying effect size and the value of nuisance parameters. Hence, we want to evaluate the one‐stage design, a two‐stage group‐sequential design, as well as a two‐stage hybrid approach that chooses between these two options based on the results of a blinded interim analysis.

#### 2.2.1 Single‐Stage Design

The simplest study design is the single‐stage design, where hypothesis testing is conducted only after all participants $n_{i},i=A,B$, in both the intervention and control groups have been recruited and followed up. Once the data collection is complete, the log‐rank test statistic in (1) is computed based on all available data. The one‐sided null hypothesis is rejected if $LR>\Phi^{-1}\left(1-\alpha \right)$, as LR is asymptotically standard normally distributed. Here, $\Phi^{-1}\left(\cdot \right)$ denotes the inverse of the cumulative distribution function of a standard normal distribution. Note that quadratic forms of the test statistic LR, which asymptotically follow a $\chi_{1}^{2}$ distribution, also exist. However, the test statistic as given in formula (1) aligns better with the group‐sequential approach (Wassmer and Brannath 2016).

#### 2.2.2 Group‐Sequential Design

As opposed to single‐stage designs, group‐sequential trial designs allow for an early stop of the trial by formally incorporating interim analyses into the trial course. During an unblinded interim analysis, the test statistic $LR_{1}$ is computed as described in Section 2.1. Let $c_{e,1}$ and $c_{f,1}$ represent the efficacy and futility boundaries, respectively. Then, the trial terminates early for efficacy if $LR_{1}\in \left(c_{e,1},\infty \right)$, and it concludes early for futility if $LR_{1}\in \left(-\infty ,c_{f,1}\right)$. Otherwise, if $LR_{1}\in \left[c_{f,1};c_{e,1}\right]$, the trial continues to the second stage. The null hypothesis is rejected after the second stage if $LR_{1+2}>c_{e,2}$, where $c_{e,2}$ denotes the critical value at the final analysis.

Due to the interim analysis, a multiple testing problem is inherent to group‐sequential methods. Therefore, the boundaries $\left\{c_{f,1},c_{e,1},c_{e,2}\right\}$ must be determined such that the probability of committing a type‐1 error at any of the analyses equals the nominal significance level α. Several well‐known methods have been developed for computing group‐sequential boundaries, for example, by Pocock (1977) and O'Brien and Fleming (1979). Throughout the whole paper, we will focus on the boundaries introduced by Pocock (1977) for illustrative purposes.

In time‐to‐event analyses, the information fraction at interim $I_{1}$ is defined as the expected number of events observed at interim relative to the planned total number of events, rather than the fraction of the target sample size accrued. Let d denote the total number of events required to achieve a target power of 1−β at an assumed hazard ratio of $\theta_{a}<1$ in a study with a recruitment duration $t_{\text{rec}}$, a follow‐up period $t_{\text{follow-up}}$, and an interim analysis planned to be conducted at calendar time $t_{\text{interim}}$. Then, the overall expected number of events at the interim analysis, $d_{1}$, can be estimated and the information fraction is calculated as $I_{1}=d_{1}/d$ for planning purposes. Regarding futility, we fix $c_{f,1}:=0$ for the remainder of the paper to indicate a stop for futility whenever the point estimate of the hazard ratio at interim, ${\hat{\theta }}_{1}$, suggests an unfavorable direction, that is, ${\hat{\theta }}_{1}>1$. Hence, we consider a binding futility stop. Using the asymptotic bivariate normality stated in (2) and setting $c_{f,1}=0$, Pocock boundaries can be determined numerically under the constraint $c_{e,1}=c_{e,2}$. For this purpose, we use the R package rpact by Wassmer and Pahlke (2023)

#### 2.2.3 New Proposal: A Hybrid Approach

In our present work, we aim to propose a new design that combines the advantages of both the always‐blinded single‐stage design and the interim‐unblinded group‐sequential design. We refer to this design as the *hybrid design* henceforth, as it serves as a compromise between the two approaches discussed so far. The hybrid design operates as follows: During the interim analysis, the study team computes the overall number of events, denoted as $d_{1}$. This number serves as a criterion for deciding whether to unblind the study. Boundaries $d_{l}$ and $d_{u}$ will be established to guide the decision regarding unblinding:

- *Group‐sequential design after interim analysis:*
  – **If** $d_{1}<d_{l}$: Unblinding may be justified when the overall number of events at the interim analysis is small, as this can hinder the detection of relevant effects. Even if a meaningful effect exists, the limited number of events may not provide sufficient statistical power to demonstrate it. In this scenario, unblinding may be valuable for conducting a futility analysis. Additionally, a surprisingly small overall event rate at interim could indicate an unexpectedly effective treatment. If this is the case, unblinding may also be warranted to assess the potential for early stopping for efficacy. Note also that unblinding in the case of a low number of events may be warranted to assess study feasibility in terms of meeting the target number of events.
  – **If** $d_{1}>d_{u}$: Unblinding may be justified in cases where there is a large number of events, as this can provide sufficient statistical power to detect an existing effect and allow for early stopping for efficacy. If the large number of events is primarily driven by an ineffective or even harmful treatment, unblinding in this context could also present an opportunity to stop the trial for futility. Moreover, a large number of events may also justify unblinding for safety monitoring.
- *Stay blinded after interim analysis:*
  – **If** $d_{l}\leq d_{1}\leq d_{u}$: In this case, the observed number of events is neither unexpectedly low nor high. Remaining blinded is motivated by the fact that the observed data are in line with the initial planning assumptions.

**FIGURE 1:**
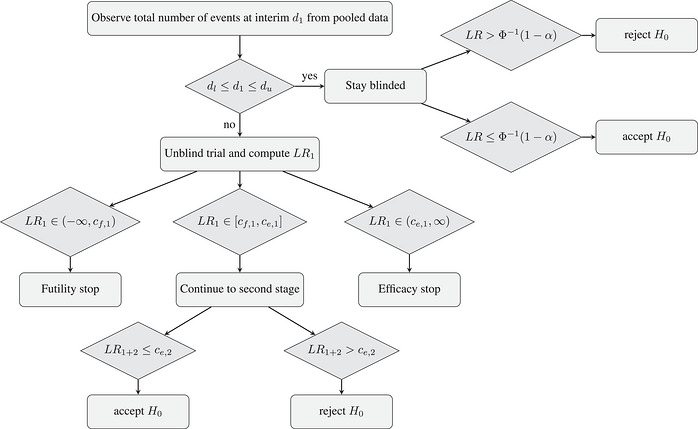
Schematic illustration of hybrid design. Note that in case of staying blinded, $LR=LR_{1+2}$; however, the notation LR is used to emphasize that no unblinded interim analysis is performed.

The design is also schematically illustrated in Figure 1. That is, if $d_{1}\notin \left[d_{l},d_{u}\right]$, the design reduces to a group‐sequential design characterized by the boundaries $\left\{c_{f,1},c_{e,1},c_{e,2}\right\}$ as presented in Section 2.2.2. In contrast, if $d_{1}\in \left[d_{l},d_{u}\right]$, it simplifies to a single‐stage design with critical value $\Phi^{-1}\left(1-\alpha \right)$ at the trial end. Clearly, the question of how to determine or choose the boundaries $d_{l}$ and $d_{u}$ remains. In the following, we introduce three heuristics that can be used to guide the selection of these parameters. The related designs will be referred to as hybrid designs I, II, and III.

#### 2.2.4 Hybrid Design I: Deriving the Boundaries $d_{l}$ and $d_{u}$ From the Assumed Interim Power

To motivate hybrid design I, we adapt the notation used by Dang et al. (2020). Let T denote the random survival time with density f(t), survival function

$$\begin{array}{c} S\left(t\right)=P\left(T>t\right)=\int_{t}^{\infty }f\left(u\right)\;du, \end{array}$$

and hazard function h(t). Let C denote the independent censoring time with density g(t) and censoring distribution function H(t)=P(C>t). It is important to note that both random variables T and C refer to the pooled sample in the context of our clinical trial. Observed survival outcomes are given by vectors $\left(X_{i},\delta_{i}\right)$ for i=1,…,n, where $X_{i}=\min\left(T_{i},C_{i}\right)$ and $\delta_{i}=1_{\left\{T_{i}\leq C_{i}\right\}}$ indicate if the event occurred before censoring. The probability of observing an event can then be expressed as

(3) $$\begin{array}{c} \int_{0}^{\infty }f\left(t\right)H\left(t\right)\;dt, \end{array}$$

representing the probability of an event occurring, provided the subject has been at risk for all t and not been censored by that time. Now, assume that patients enter the study at random entry times $E_{i}$ with cumulative distribution $Q_{E}\left(t\right)=P\left(E_{i}\leq t\right)$, where $Q_{E}\left(t\right)=0$ for t<0, and let $E_{i}$ be independent of $T_{i}$ and $C_{i}$. Then, for a fixed analysis time L, the expected number of observed events in a sample of size n is

(4) $$\begin{array}{c} D_{L}=n\underset{:=p_{\text{event}}}{\underset{︸}{\int_{0}^{\infty }f\left(t\right)H\left(t\right)Q_{E}\left(L-t\right)\;dt}}=n\cdot p_{\text{event}}, \end{array}$$

where $Q_{E}\left(L-t\right)=0$ for t>L. Thus, the factor $Q_{E}\left(L-t\right)$ weights the event probability $\int_{0}^{\infty }f\left(t\right)H\left(t\right)dt$ by the likelihood that a subject has already been recruited and is therefore at risk at the analysis time L; see also Dang et al. (2020). Note that the integral above can be computed either analytically or must be evaluated via numerical integration, depending on the assumptions regarding survival and censoring behavior (Friede et al. 2019). For details in the case of exponentially distributed survival and censoring event times as well as uniform recruitment, we refer to Rubinstein et al. (1981) or Lakatos and Lan (1992). For sample size planning, the Schoenfeld formula

(5) $$\begin{array}{c} d=\frac{4\cdot {\left(z_{1-\alpha }+z_{1-\beta }\right)}^{2}}{\log{\left(\theta_{a}\right)}^{2}} \end{array}$$

is often used, here for a one‐to‐one allocation ratio and quantiles z of the standard normal distribution (Schoenfeld 1983). The number of events d refers to events of the intervention and control group combined and assures a power of 1−β at a one‐sided significance level α and an assumed hazard ratio of $\theta_{a}$. From the Schoenfeld formula in Equation (5), we can obtain that the power for a specific hazard ratio $\theta_{a}$ at a given number of events d can be approximated by

$$\begin{array}{cc} \text{Power} & =\Phi \left(\sqrt{\frac{1}{4}\cdot d\cdot \log{\left(\theta_{a}\right)}^{2}}-\Phi^{-1}\left(1-\alpha \right)\right). \end{array}$$

Replacing d by the expected number of events $D_{L}$ at analysis time L as well as α by the type‐1 error rate spent at interim $\alpha_{1}$, we obtain an expected interim power of

(6) $$\begin{array}{cc} \text{Power} & =\Phi \left(\sqrt{\frac{1}{4}\cdot D_{L}\cdot \log{\left(\theta_{a}\right)}^{2}}-\Phi^{-1}\left(1-\alpha_{1}\right)\right). \end{array}$$

From Equation (6), we can calculate the expected power at the interim analysis based on our initial planning assumptions and the anticipated survival behavior. It may be reasonable to consider unblinding if, given $\theta_{a}$, the interim power is substantially larger or smaller than expected, for instance, if it deviates by $\pm \Delta_{1}\%$ from the initial assumption. Based on this reasoning, we can determine the boundaries $d_{l}$ and $d_{u}$ by solving the equation

$$\begin{array}{cc} & \Phi \left(\sqrt{\frac{1}{4}\cdot D_{L}\cdot \log{\left(\theta_{a}\right)}^{2}}-\Phi^{-1}\left(1-\alpha_{1}\right)\right)\mp \Delta_{1}\\ & \overset{!}{=}\Phi \left(\sqrt{\frac{1}{4}\cdot d_{l,u}\cdot \log{\left(\theta_{a}\right)}^{2}}-\Phi^{-1}\left(1-\alpha_{1}\right)\right), \end{array}$$

for $d_{l}$ and $d_{u}$, resulting in

dl=4log(θa)2Φ−1Φ14DLlog(θa)2−Φ−1(1−α1)14DLlog(θa)2−Φ−1(1−α1)−Δ1+Φ−1(1−α1)214DLlog(θa)2−Φ−1(1−α1),du=4log(θa)2Φ−1Φ14DLlog(θa)2−Φ−1(1−α1)14DLlog(θa)2−Φ−1(1−α1)+Δ1+Φ−1(1−α1)214DLlog(θa)2−Φ−1(1−α1),

where ⌊c⌋ and ⌈c⌉ denote that the lower boundary will be rounded down to the nearest integer, and the upper boundary will be rounded up to the nearest integer, ensuring that unblinding corresponds to a deviation of at least $\Delta_{1}$ in interim power. Note that, since $\alpha_{1}$ depends on the underlying group‐sequential design, the selection of $\Delta_{1}$ as well as the identification of its admissible region (the values of $\Delta_{1}$ that produce valid stopping boundaries) should be guided by the configuration of the study design. Details on the choice of $\Delta_{1}$ can be found in Appendix A.1.

#### 2.2.5 Hybrid Design II: Deriving the Boundaries $d_{l}$ and $d_{u}$ Based on the Unblinding Probability

For hybrid design I, we have stated that we can compute the probability of an event occurring by time L for all participants $X_{i}$, where i=1,…,n. Our model also assumes the independence of all participants, which allows us to represent the probability for each individual $X_{i}$ as a Bernoulli distribution, specifically, $X_{i}\overset{iid}{∼}B\left(p_{\text{event}}\right)$, where $p_{\text{event}}$ denotes the event probability as defined in Equation (3). Consequently, the random variable

$$\begin{array}{c} Y:=\sum_{i=1}^{n}X_{i}∼\text{Bin}\left(n,p_{\text{event}}\right) \end{array}$$

follows a binomial distribution with an expected value given by $E\left[Y\right]=D_{L}$. Based on this assumption, the second hybrid design aims to determine the thresholds $d_{l}$ and $d_{u}$ as quantiles derived from the underlying binomial distribution. Specifically, we can establish $d_{l}$ such that the probability of unblinding the trial due to a small number of events equals a predetermined probability of $\Delta_{2}$. This can be expressed as follows:

$$\begin{array}{c} P\left(Y<d_{l}\right)\overset{!}{=}\Delta_{2}\;⟺\;d_{l}=F_{Y-}^{-1}\left(\Delta_{2}\right), \end{array}$$

where $F_{Y-}^{-1}$ denotes the inverse left‐continuous cumulative distribution function of Y. Similarly, $d_{u}$ can be determined as

$$\begin{array}{c} P\left(Y>d_{u}\right)\overset{!}{=}\Delta_{2}\;⟺\;d_{u}=1-F_{Y+}^{-1}\left(\Delta_{2}\right), \end{array}$$

where $F_{Y+}^{-1}$ represents the inverse right‐continuous cumulative distribution function of Y.

#### 2.2.6 Hybrid Design III: Deriving the Boundaries $d_{l}$ and $d_{u}$ From the Deviation of the Observed From the Expected Number of Events

The concept behind hybrid design III is similarly grounded in the idea that the probability of observing an event by time‐point L can be modeled as a binomial distribution with an expected value of $D_{L}$. In this design, we directly assess deviations from the expected value $D_{L}$. Specifically, given an allowable deviation of $\Delta_{3}$, we define the thresholds as follows:

$$\begin{array}{c} d_{l,u}=D_{L}\mp \Delta_{3}D_{L}\;⟺\;d_{l,u}=D_{L}\left(1\mp \Delta_{3}\right). \end{array}$$

As before, the resulting boundaries will be rounded to the nearest integer.

### 2.3 Example for the Three Hybrid Designs

To illustrate all three hybrid designs, consider a balanced two‐arm clinical trial aiming to recruit a total of n=208 patients uniformly over a 36‐month period. All patients will be followed for a maximum of 12 months. We assume that the 1‐year event probability is $p_{A}=0.1$ for the treatment group, while the control group has a larger 1‐year event probability of $p_{B}=0.2$. Under exponentially distributed survival times, this corresponds to a hazard ratio of $\theta_{a}=0.472$. Additionally, we assume a 1‐year censoring probability of 0.01 for both arms, with censoring times also modeled using an exponential distribution.

An interim analysis is scheduled after 24 months of recruitment, where we expect to have enrolled approximately 138 patients. Under these assumptions, a two‐stage Pocock‐design with information fraction of $I_{1}=0.3$ has a power of approximately 80% at a one‐sided significance level of α=0.025. After 24 months of recruitment (L=24), the expected number of events is $D_{L}\approx 20$. By the end of the study (at L=48 months), we expect a total of 66 events, which aligns with an information fraction of $I_{1}\approx 0.3$ (≈20/66). This design allocates a type‐1 error rate of $\alpha_{1}=0.01409$ to the interim analysis. Given $D_{L}=20$, the probability of rejecting $H_{0}:\theta \geq 1$ at interim is approximately 0.30 at $\theta_{a}=0.472$.

For hybrid design I, assume that unblinding should be triggered if the interim power deviates by ±10% from the expected level. This is achieved by setting $\Delta_{1}=0.1$, resulting in unblinding boundaries of $\left[d_{l},d_{u}\right]=\left[13,28\right]$. For hybrid design II, the goal might be to limit the probability of unblinding to 20%. To achieve this, $\Delta_{2}=0.1$ is allocated for both high and low event counts, yielding an unblinding interval of $\left[d_{l},d_{u}\right]=\left[14,26\right]$. In hybrid design III, unblinding may be indicated if the observed number of events deviates by $\Delta_{3}=0.2$ from the expected count. This results in unblinding boundaries of $\left[d_{l},d_{u}\right]=\left[16,25\right]$. In this example, the unblinding boundaries are most conservative for hybrid design I and least conservative for hybrid design III. Practically, this means that hybrid design I has the lowest probability of unblinding, while hybrid design III has the highest. Put differently, the “group‐sequential path” contributes more significantly to hybrid design III, whereas the “single‐stage path” plays a larger role in hybrid design I. Clearly, the contribution of both individual designs can be calibrated via the design parameter $\Delta_{i}$. We do not aim at optimizing those boundaries in general in this paper. Instead, our goal is to choose reasonable boundaries for illustrating those new hybrid designs. However, we provide further information on the behavior of the boundaries in Appendix A.1.

## 3 Simulation Study

In our simulation, we consider a trial with the same recruitment, analyses, and follow‐up times used in Section 2.3. We consider two different sets of input assumptions (Study 1 and Study 2) as depicted in Table 1.The scenarios are selected to represent two different settings: in Study 1, the probability of an event occurring in either of the treatment arms is relatively low, while in Study 2 both probabilities are notably higher. This approach allows us to model scenarios that mimic time‐to‐event trials for both rare and more common events. Additionally, in both settings, the parameter values $\theta_{a}=0.00878/0.0186=0.471$ and $\theta_{a}=0.0426/0.0764=0.557$ are chosen to reflect a beneficial effect of the experimental compound. Exemplary Kaplan–Meier curves illustrating both settings are provided in Appendix A.5. Table 2 summarizes the reference two‐stage group‐sequential designs, detailing the required sample sizes to achieve a target power of 80%. It is important to note that the expected number of events closely aligns with the expected interim information, as $\frac{20}{66}\approx 0.3$ and $\frac{41}{105}\approx 0.4$ at the analysis time of 24 months.

**TABLE 1:** Planning assumptions on relevant survival parameters for both considered studies. The underlying distribution for the time‐to‐event as well as the time‐to‐censoring is an exponential distribution.

Group	1‐Year event		Hazard		Median survival		1‐Year censoring	
	probability		$h_{j}\left(t\right)$		time		probability	
Setting	*Study 1*	*Study 2*	*Study 1*	*Study 2*	*Study 1*	*Study 2*	*Study 1*	*Study 2*
Control	0.2	0.6	0.01860	0.0764	37.3	9.1	0.01	0.01
Treatment	0.1	0.4	0.00878	0.0426	78.9	16.3	0.01	0.01

**TABLE 2:** Parameter values for two‐stage group‐sequential reference study designs for a study with $t_{\text{rec}}=36$ and $t_{\text{interim}}=24$. The sample size n refers to the total sample size, with equal allocation across the two groups. Note that the information $I_{1}$ is approximate and the expected number of events is rounded to the closest integer.

Parameter	Study 1	Study 2
Hazard ratio $\theta_{a}=h_{V}/h_{P}$	0.472	0.557
One‐sided type‐1 error rate	0.025	0.025
Interim information $I_{1}$	0.3	0.4
Power	0.8	0.8
Type of design	Pocock	Pocock
Binding futility boundary $c_{f}$	$c_{f}=0$	$c_{f}=0$
Required sample size n	208	138
Expected number of events at interim under $H_{1}$	20	41
Expected number of events at final under $H_{1}$	66	105

The same studies were additionally planned using the frequently applied O'Brien–Fleming boundaries instead of the Pocock boundaries. Details on the corresponding design and simulation results are provided in Supporting Information 1. In the following, we aim to describe the simulation study of performance characteristics based on the ADEMP scheme (i.e. referring to aims, data‐generating mechanisms, estimands, methods, and performance measures) proposed by Morris et al. (2019).

The *aim* of our simulation study is to evaluate the performance of the hybrid designs in comparison to a single‐stage and a group‐sequential design. More concretely, we investigate the performance of our hybrid designs in trials that are initially planned as detailed above (with Study 1 and Study 2).

In the survival setting, we need to account for three distinct random processes when it comes to the *data‐generating mechanism*: the survival process, the censoring process, and the study entry times. We assume independent right‐censoring, with a 1‐year censoring probability of $p_{cens}=0.01$, corresponding to an exponential distribution with rate $h_{cens}=0.00084$ for both groups. For study entry times, we assume a uniform recruitment model, meaning participant entry times are simulated as $E_{t}\overset{\text{iid}}{∼}U\left(0,t_{\text{rec}}\right)$, where $t_{\text{rec}}=36$ months. For the survival times $T_{j}$, we similarly assume an underlying exponential distribution $T_{j}\overset{iid}{∼}Exp\left(h_{j}\left(t\right)\right),j=A,B$, with hazards being selected based on the specific study design. In terms of parameterization, we use 1‐year survival probabilities and hazard ratios, from which we calculate the hazard rates for both groups based on the exponential distribution.

- In Study 1, we aim to recruit n=208 participants over $t_{\text{rec}}=36$ months. To account for variability in recruitment, we model the monthly recruitment as a random variable $R_{t}\overset{\text{iid}}{∼}P\text{oi}\left(208/36\right)$ so that $E\left[\sum_{t=1}^{36}R_{t}\right]=\sum_{t=1}^{36}E\left[R_{t}\right]=36\cdot \frac{208}{36}=208$. For assessing the impact of different effects, we consider the following scenarios:
  (a) We vary the assumed 1‐year event probability in the control group by setting $p_{B}=\left(0.05,0.1,0.2,0.4,0.6\right)$, which corresponds to hazard rates $h_{B}=\left(0.0043,0.0088,0.0186,0.0426,0.0763\right)$. For the treatment group, we calculate the hazard $h_{A}$ to maintain a constant hazard ratio of θ=0.472, yielding $h_{A}=\left(0.0020,0.0041,0.0088,0.0201,0.0360\right)$. This scenario examines the operating characteristics based on the number of events while keeping the hazard ratio fixed at the value specified as planning assumptions.
  (b) We examine the effects of varying the hazard ratio, setting θ=(0.2,0.3,0.4,0.47,0.5,0.6,0.7,0.8,0.9,1,1.1) while fixing $p_{B}=0.2$. In this case, we simulate exponential survival times with parameters $h_{B}=0.0186$ and $h_{A}=\left(0.0037,0.0056,0.0074,0.0088,0.0093,0.0112,0.0130,0.0149,0.0167,0.0186,0.0205\right)$. This simulation assesses how varying the hazard ratio affects operating characteristics as the total number of events changes. Note that for θ=1, we simulate under the null hypothesis.
  This eventually yields five settings for (a) and eleven settings for (b), that is, 16 settings in total.
- In Study 2, the recruitment goal over 36 months is n=138. To again account for variability in recruitment, we model the monthly recruitment as a random variable $R_{t}\overset{\text{iid}}{∼}P\text{oi}\left(138/36\right)$. For assessing effects, we consider the following scenarios similarly to Study 1:
  (c) We vary the assumed 1‐year event probability in the control group by setting $p_{B}=\left(0.1,0.2,0.4,0.6,0.7,0.8\right)$, which corresponds to hazard rates $h_{B}=\left(0.0088,0.0186,0.0426,0.0763,0.1003,0.1341\right)$. For the treatment group, we calculate the hazard $h_{A}$ to maintain a constant hazard ratio of θ=0.557, yielding $h_{A}=\left(0.0049,0.0104,0.0237,0.0425,0.0559,0.0747\right)$.
  (d) We examine the effects of varying the hazard ratio, setting θ=(0.2,0.3,0.4,0.5,0.557,0.6,0.7,0.8,0.9,1,1.1) while fixing $p_{B}=0.6$. In this case, we simulate exponential survival times with parameters $h_{B}=0.0763$ and $h_{A}=\left(0.0153,0.0229,0.0305,0.0382,0.0425,0.0458,0.0534,0.0611,0.0687,0.0763,0.0840\right)$.
  This eventually yields six settings for (c) and 11 settings for (d), that is, 17 settings in total.

We set the number of to $n_{sim}=10^{\prime }000$.

Now we come to the description of the *estimands and performance measures*. In our simulation study, we investigate different parameters relating to testing, study design performance, and estimation. Our estimands of interest as well as corresponding estimators or empirical performance characteristics are outlined in Table 3.

**TABLE 3:** Estimands and estimators considered in our simulation study.

Domain	Estimand	Estimator/performance characteristics
Testing	Type‐1 error rate $P_{H_{0}}\left(\text{reject}\;H_{0}\right)$	$\frac{1}{n_{sim}}\sum_{i=1}^{n_{sim}}1_{\left\{\text{Rej.}\;H_{0}|H_{0}\right\}}$
	Power $P_{H_{1}}\left(\text{reject}\;H_{0}\right)$	$\frac{1}{n_{sim}}\sum_{i=1}^{n_{sim}}1_{\left\{\text{Rej.}\;H_{0}|H_{1}\right\}}$
Study design	Expected sample size	$\frac{1}{n_{sim}}\sum_{i=1}^{n_{sim}}N_{i}$
		with $N_{i}$ being the simulated sample size in iteration i
	Expected trial duration	$\frac{1}{n_{sim}}\sum_{i=1}^{n_{sim}}T_{i}$
		with $T_{i}$ being the simulated trial duration in iteration i
	Probability of unblinding	$\frac{1}{n_{sim}}\sum_{i=1}^{n_{sim}}1_{\left\{d_{1}\notin \left[d_{l},d_{u}\right]\right\}}$
Estimation	Relative bias of $\hat{\theta }$	$\frac{100}{\theta }\cdot \left(\frac{1}{n_{sim}}\sum_{i=1}^{n_{sim}}\hat{\theta }-\theta \right)$

Note that the definitions of some estimands, such as the power and type‐1 error rate, are dependent on the method under investigation. Details on the performance characteristics depending on the study design under investigation are provided in Table A1 in Appendix A.2.

**TABLE 4:** Boundaries $\left\{d_{l},d_{u}\right\}$ for all hybrid designs in the example clinical trial. For all designs, the parameters $\Delta_{i}$ were the same as in the simulation study.

Design	Deviation parameter	$d_{l}$	$d_{u}$
Hybrid design I	0.1	8	35
Hybrid design II	0.1	15	27
Hybrid design III	0.3	17	26

Regarding *methods*, each simulated dataset will be analyzed using all the presented study designs: the single‐stage design, the group‐sequential design, and the three hybrid design candidates. As in Section 2, for hybrid design I, unblinding is triggered if the interim power deviates by ±10% from the expected level. For hybrid design II, the probability of unblinding is limited to 20%, with 10% allocated for high event counts and 10% for low event counts. For hybrid design III, unblinding will occur if the observed number of events deviates by ∓20% from the expected number. Hence, the following design boundaries $\left\{d_{l};d_{u}\right\}$ arise

- Hybrid design I: {13;28} in Study 1 and {30;54} in Study 2,
- Hybrid design II: {14;26} in Study 1 and {34;49} in Study 2, and
- Hybrid design III: {16;25} in Study 1 and {33;51} in Study 2.

Note that because the assumed 1‐year event probabilities are higher in the second study, the boundaries $d_{l}$ and $d_{u}$ are generally at higher levels.

Due to the “pretest–test” situation induced by the new hybrid designs, we also added a small simulation study regarding the type‐1 error rate, which can be found in Appendix A.3.

## 4 Results

Note that the results of the small simulation study evaluating the type‐1 error rate can be found in Appendix A.3. For θ=1, all methods adhere to the target significance level of α=0.025.

**FIGURE 2:**
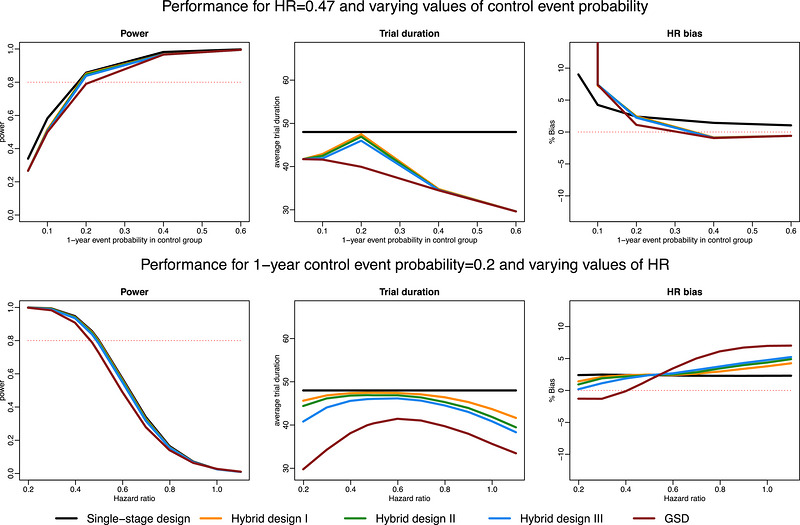
Power, expected trial duration, and relative bias of the hazard ratio in Study 1.

**FIGURE 3:**
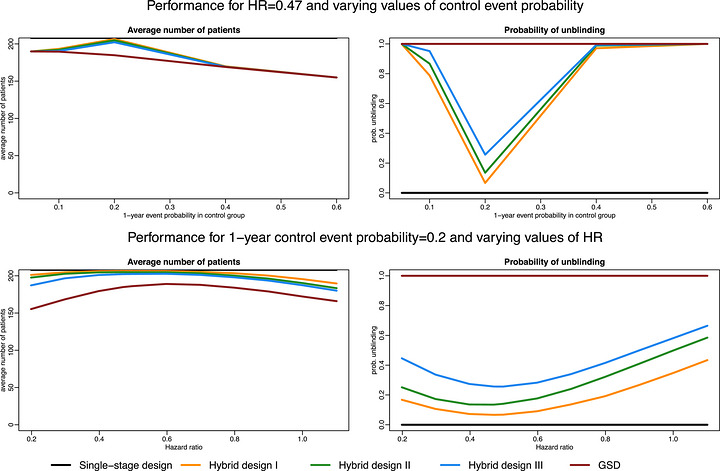
Expected sample size and probability of unblinding in Study 1.

In this section, we begin by presenting the simulation results from *Study 1* as given in Figures 2 and 3. In the upper row of the results, the hazard ratio is fixed while the 1‐year event probability in the control group is varied. Conversely, in the lower row, the hazard ratio is varied while keeping the 1‐year event probability in the control group fixed.

For both settings, we observe that the single‐stage design achieves the highest power, while the classical group‐sequential design exhibits the lowest power. This difference arises because group‐sequential methods need a multiplicity adjustment due to the interim analysis. Regarding the hybrid designs, we observe that their power consistently lies between that of the single‐stage design and the group‐sequential design, reflecting that they are constructed to be a compromise between these two study design approaches. Similar patterns are observed regarding expected trial duration and expected sample size. While the single‐stage design maintains a fixed sample size, the group‐sequential designs achieve the lowest expected sample size due to the possibility of early stopping for efficacy or futility. For the hybrid designs, an additional criterion for unblinding is introduced. Consequently, the hybrid designs are positioned between the single‐stage and group‐sequential designs in terms of expected sample size and trial duration as well. Regarding bias of the hazard ratio, we draw attention to the lower‐ and upper‐right plots. For hazard ratios below 0.5, the group‐sequential design initially exhibits a slight negative bias, whereas the other designs show a positive bias. Overall, however, the group‐sequential design tends to yield the smallest deviation between the estimated and true hazard ratios. For hazard ratios greater than 0.5, the group‐sequential design has the largest bias, while the hybrid designs exhibit bias levels between those of the single‐stage and group‐sequential designs, with the single‐stage design showing the smallest bias. Although the bias in the hazard ratio estimated from a single‐stage design using a standard Cox proportional hazards model may seem surprising, it can be explained by the fact that the Cox model provides an unbiased estimate of the logarithm of the hazard ratio. Applying the exponential function, which is a convex transformation, then introduces a slight positive bias according to Jensen's inequality. Note also that the observed bias aligns with findings in the literature (Johnson et al. 1982). A more detailed investigation of the bias under $H_{0}$ can be found in Appendix A.4, illustrating that the bias in the single‐stage design plateaus at a low level as the number of events increases.

Lastly, we compare the average number of patients and the probability of unblinding among the three candidate hybrid designs (cf. Figure 3). The expected savings in sample size for the three hybrid designs are, once again, slightly less pronounced than with the group‐sequential design, as stopping at interim requires meeting an additional criterion. Furthermore, the savings in expected sample size (similar to those in trial duration) are most significant for extreme hazard ratios. For example, as shown in the plot on the bottom left, with hazard ratios around 0.2, the group‐sequential design is expected to save approximately 50 patients, whereas hybrid designs II and III still achieve expected savings of 10–25 patients. Consistent with the boundaries specified in Section 3, hybrid design III exhibits the most aggressive unblinding boundaries, resulting in the highest probability of unblinding at interim. As expected, this observation holds true regardless of the underlying parameterization. Beyond the raw probability of unblinding, it is also interesting to consider the probability of early stopping following unblinding, as well as the probability of continuing the trial with a group‐sequential design when the hybrid design remains blinded. Illustrative results for these quantities are provided in Appendix A.7. Across the scenarios and designs considered, the estimated probability of stopping at an interim analysis after unblinding ranges from 0.26 to 0.98, while the estimated probability of continuing after remaining blinded ranges from 0.01 to 0.84, excluding cases where the hybrid designs always unblind. These results suggest that whether studies are unblinded for interim monitoring strongly depends on the underlying treatment effects and the specific design, which is governed by the unblinding boundaries. For extreme hazard ratios such as θ=0.2, where the group‐sequential design would always stop, the hybrid design may not unblind because of the limited number of events. Thus, while hybrid designs help prevent unnecessary unblinding—thereby saving resources, preserving trial integrity, and minimizing bias in effect estimates—they also carry the trade‐off of occasionally failing to unblind in situations where the group‐sequential design would have led to an interim stop. In conclusion, the hybrid design's construction as a compromise between a never‐unblind and always‐unblind design is reflected in its performance measures.

Since the simulation results for *Study 2* are largely consistent with those of *Study 1*, we provide only a brief description here and refer to the corresponding plots in Appendix A.6 (Figures A8 and A9). It is worth noting that, in Study 2, the unblinding probability is highest for hybrid design II, rather than hybrid design III, resulting in the largest savings in time and expected sample size for this design among all hybrid designs. This highlights that, even with fixed deviation parameters $\Delta_{i}$, the probability of unblinding depends on the specific underlying study design. However, this outcome is clearly influenced by the choice of deviation parameters, which should, in practice, be carefully determined during the planning phase (cf. also Section A.1 in the Appendix).

Results from the simulation study using O'Brien–Fleming boundaries instead of Pocock boundaries are presented in Supporting Information 2. While broadly similar to the Pocock case, the benefits of the hybrid designs are slightly reduced due to the stricter efficacy criterion of the O'Brien–Fleming design. Apart from this, the performance characteristics still represent a compromise between the fixed and group‐sequential designs, although the gap between them is narrower, as continuation to the planned trial end is more likely. Moreover, one also has to keep in mind the underlying inflation factors. The inflation factor associated with O'Brien–Fleming boundaries is lower than for the Pocock boundaries and almost equals the value one in our two‐stage design. Therefore, the power gain from not performing an unblinded interim analysis is almost negligible when using O'Brien–Fleming boundaries. Hence, the hybrid design might be more suitable for designs that consider boundaries associated with a larger inflation factor.

## 5 Clinical Trial Example

In order to illustrate our hybrid designs in a real‐world example, we draw inspiration from Love et al. (2015), who conducted a breast cancer trial. Specifically, their study investigated the effect of surgical timing during the menstrual phase on disease‐free survival. During the conduct of this trial, concerns arose that the initially assumed failure probabilities were too high, as new evidence from other trials became available (see Hade et al. 2019). Since extending the follow‐up time was not a viable option, the researchers employed a blinded sample size re‐estimation approach (Hade et al. 2010). More concretely, they defined the blinded estimate of the survival function $S_{blinded}\left(t\right)$ as the average of the survival functions from both groups, that is, $S_{blinded}\left(t\right)=\frac{1}{2}\left(S_{f}{\left(t\right)}^{HR}+S_{f}\left(t\right)\right)$, where $S_{f}\left(t\right)$ denotes the survival function of the control group and $S_{f}{\left(t\right)}^{HR}$ that of the treatment group under a target hazard ratio. Given that $S_{blinded}\left(t\right)$ can be estimated in a blinded manner, they proposed estimating $S_{f}\left(t\right)$ and $S_{f}{\left(t\right)}^{HR}$ either through parametric assumptions (see also Friede et al. 2019) or by combining the observed data with prior information, and then recomputing the sample size based on the updated hazard ratio. By showing that the type‐1 error rate remains well controlled, the authors demonstrate that this strategy ensured that the overall study integrity and confidence in the trial results were preserved. When faced with similar uncertainties at the planning stage, yet wishing to avoid an unblinded interim analysis, one could alternatively consider one of the hybrid approaches we proposed within this article. In this specific trial, the researchers planned to analyze the data using a log‐rank test. The trial was designed with a target power of 80% and a two‐sided significance level of 5%. They accounted for a 2–3% loss to follow‐up, assuming an initial hazard ratio of 0.58. We assume that the hazard ratio is based on 1‐year event rates of $p_{A}=0.41$ in the intervention group and $p_{B}=0.6$ in the control group. It should be noted that these assumptions are chosen for illustrative purposes and are not necessarily the same as those used in the original trials. The trial was planned to recruit participants over a period of 24 months, followed by an additional 48 months of follow‐up, as outlined in Hade et al. (2019). This results in a total study duration of 72 months for the final analysis. Given that the majority of the trial duration is dominated by follow‐up time, a late interim analysis would primarily allow for saving time but not for reducing the required sample size. Assuming that the investigators prioritize sample size reductions, an early interim analysis is therefore incorporated approximately 12 months after the start of the recruitment phase. This will be conducted using a Pocock design, featuring a binding futility boundary at $c_{f,1}=0$ and critical values $c_{e,1}=c_{e,2}=2.191$. For these parameter values, a sample size of approximately n=152 participants and around d=141 events will be required to achieve a target power of 80%. Based on these planning assumptions, the expected number of events at interim was approximately 21. The corresponding hybrid designs are summarized in Table 4.

Table 4 highlights the substantial variability in interim boundaries across the different heuristic designs, depending on the criteria for unblinding. Using these planning assumptions, we simulated an exemplary dataset. In this simulated dataset, a total of n=82 participants were recruited after 12 months of recruitment, with 27 of them having already experienced the event of interest (occurrence of breast cancer). This leads to an unblinding decision in hybrid design III, as 26<27. However, for hybrid design I and II, the trial remains blinded. With an estimated hazard ratio of ${\hat{\theta }}_{1}=0.58$, the interim test statistic is calculated as $LR_{1}=1.39$. Since $c_{f,1}=0.0<LR_{1}<c_{e,1}=2.191$, the group‐sequential design as well as the unblinded hybrid design III do not meet the criteria for early stopping due to efficacy, and recruitment continues until month 24. After an additional follow‐up period of 48 months, that is, at 72 months, the second analysis is conducted. At this point, all n=152 patients have been recruited and 140 events have been observed. Correspondingly, the log‐rank test statistic is $LR_{1+2}=4.26>c_{e,2}=2.191$, such that the group‐sequential design and the unblinded hybrid design III lead to a rejection of the null hypothesis. Accordingly, hybrid designs I and II as well as the single‐stage design reject the null hypothesis $H_{0}:\theta \geq 1$ at a one‐sided α=0.025 since $LR_{1+2}=4.26>1.96$.

## 6 Discussion

In this paper, we have presented a new statistical method, which combines the advantages of blinded and unblinded adaptive designs with a focus on time‐to‐event endpoints. The core concept of the design is to introduce an additional criterion for unblinding with the goal of ensuring unblinding occurs only when there is a justification to do so. In general, the new hybrid design type can also be applied to other types of endpoints. We presented three versions of the hybrid design. The versions differ in terms of how the unblinding boundaries are derived, namely based on the interim power, on the unblinding probability, and on the deviation between the observed and expected number of events.

The new design type was evaluated by an extensive simulation study described according to the ADEMP scheme. We paid close attention to the power values and actual type‐1 error rate due to the “pretest–test” alike situation. Moreover, we used performance measures such as the expected trial duration, the expected sample size, the probability of unblinding, and the relative bias of our hazard ratio. We have demonstrated that the type‐1 error rate is adequately controlled for the new hybrid designs. Our analyses with respect to the other performance parameters were divided into two different settings: In Study 1, the event probability was very low in both the treatment and control groups. In Study 2, the probabilities of events were assumed to be higher. We observed that the power of the hybrid designs lies between the one of a single‐stage design and the one of the classical group‐sequential design. Similar results were obtained for the expected sample size and trial duration. In line with the literature, we have also found that for small values of the true hazard ratio, the group‐sequential design has a negative bias. The other designs considered showed a small positive bias, which follows from applying the exponential function, that is, a convex transformation, to the logarithm of the hazard ratio. For hazard ratios of 0.5 and larger, the fixed design had the smallest bias, followed by the hybrid design, and the classical group‐sequential design showed the largest bias. The larger the number of events became, the bias plateaued at a low level. This is in line with the known asymptotic behavior of the hazard ratio. Moreover, we have seen that the probability of unblinding depends both on the choice of the deviation parameters $\Delta_{i}$ and the underlying study design.

We illustrated the application of the new hybrid design with a clinical trial example. The example highlights how the hybrid design can address a current gap in trial planning: While a blinded interim analysis is preferred, assumed effect size estimates can potentially be too pessimistic or too optimistic. This issue can only be addressed by unblinding the trial. The hybrid design offers an in‐between solution. It is not generally opened up for unblinding, but it provides the possibility while controlling the type‐1 error rate. The three variants of the hybrid design led to different upper and lower boundaries and consequently to different decisions whether to unblind or not. Note that in all simulations, we assumed independent right‐censoring as well as left‐truncation, that is, the entry times are simulated independently from the event times.

In real‐life application scenarios, one needs to think about the minimal number of events required. Moreover, we always tested for efficacy even if the number of events is very low. For the sake of simplicity, we only considered binding futility stopping boundaries in the simulations and assumed that the observed information matches the planning assumptions. In practice, however, the methods are also applicable to non‐binding futility boundaries. Moreover, if unblinding occurs at the interim analysis, we recommend recalculating the boundaries in response to the observed information fraction using an α‐spending approach.

**FIGURE A1:**
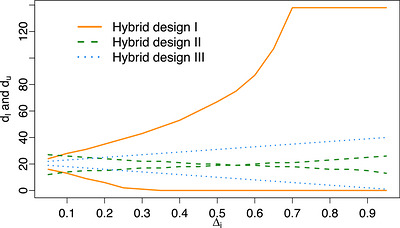
Hybrid design boundaries in the example of Section 2.3 depending on the choice of $\Delta_{i}$.

**FIGURE A8:**
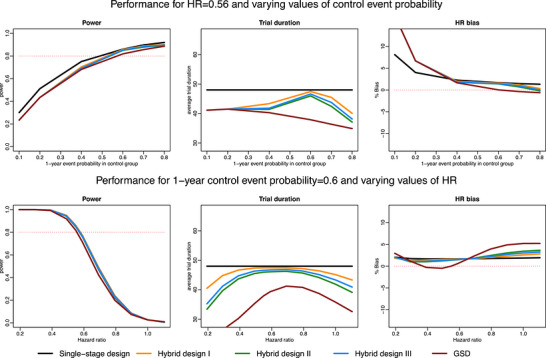
Power, expected trial duration, and relative bias of the hazard ratio in Study 2.

**FIGURE A9:**
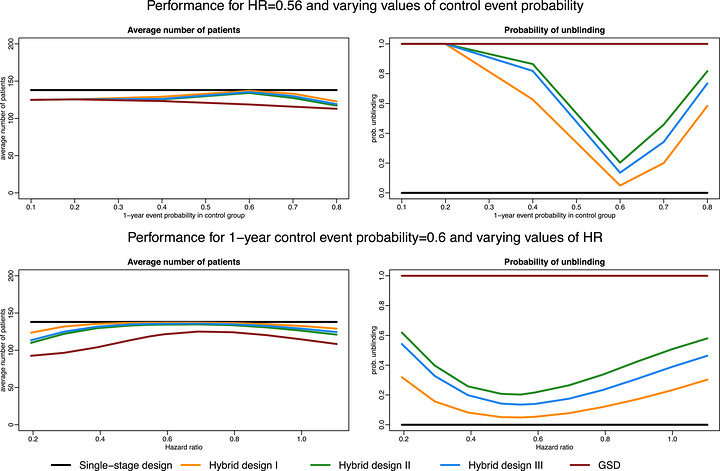
Expected sample size and probability of unblinding in Study 2.

For practical application, one should also note that for the decision to unblind, it should be considered that stopping for futility or efficacy should not be overly unlikely. This means that the interim stopping boundaries should not be too conservative in either direction; otherwise, unblinding is unlikely to provide any benefit with respect to early trial termination. Therefore, we recommend a thorough evaluation of the choice of boundaries $d_{l}$ and $d_{u}$ during the trial planning in dependence on the deviation parameters $\Delta_{i}$. We suggested three heuristic approaches, but other variations are clearly also possible. Note that our goal was not to optimize the choice of the boundaries $\left\{d_{l};d_{u}\right\}$ and that specific value limitations, for example, as seen for hybrid design I in Figure A1, need to be kept in mind at the planning stage of a trial. While the hybrid approaches may avoid the complexities of unblinding a trial in situations where a correspondingly planned group‐sequential design would have led to trial continuation, it may also result in remaining blinded when the group‐sequential design would have prompted early discontinuation for any reason, as illustrated in Table A2 in Appendix A.7. Beyond the heuristic designs presented here, future designs could aim to calibrate the boundaries so as to maximize the probability of “identifying the best designs” for interim monitoring. It should be emphasized, however, that unblinding decisions should not be driven solely by considerations of futility or efficacy; they may also be motivated by safety or feasibility, particularly in cases with an unexpectedly large or small number of events, regardless of whether a group‐sequential design would have led to the same decision. This highlights the importance of carefully calibrating the unblinding thresholds in advance, ideally in collaboration with the clinical team.

The proposed method provides a motivation for various new research questions. For example, interesting points to evaluate in the future would be to consider the difference of interim time points based on calendar times versus the number of events required for defining an interim analysis, as well as fixed versus flexible follow‐up times. Moreover, one may vary the number of interim analyses, consider competing risks and extend the method to baseline covariate adjustment. Furthermore, combining the hybrid design with (blinded) sample size recalculation can address the uncertainty about the underlying effect size and/or nuisance parameters even more directly. Moreover, as we focused on introducing the framework in general, we used the inflation factor as in a group sequential design for sample size planning to ensure a large enough power. In the future, also other values of the inflation factor could be worth considering, which provide a reasonable in‐between solution of a fixed design and a group‐sequential design.

Overall, we have added a new type of adaptive group sequential study design where unblinding is only conducted if deemed necessary based on interim study data.

## Conflicts of Interest

The authors declare no conflicts of interest.

## Open Research Badges

This article has earned an Open Data badge for making publicly available the digitally‐shareable data necessary to reproduce the reported results. The data is available in the Supporting Information section.

This article has earned an open data badge “**Reproducible Research**” for making publicly available the code necessary to reproduce the reported results. “The results reported in this article could fully be reproduced.”

## Supporting information


**Supporting File 1:** bimj70151‐sup‐0001‐SuppMat.pdf.

**Supporting File 2:** bimj70151‐sup‐0002‐Datacode.zip.

## Figures and Tables

**FIGURE A2:**
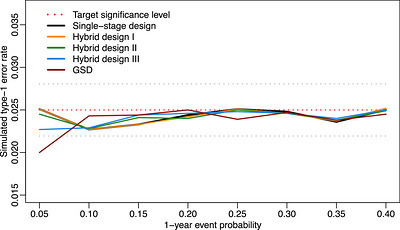
Simulated type‐1 error rates for n=180 (nominal level α=0.025, one‐sided). For hybrid design I: $\theta_{a}=0.5$ was used to derive the interim unblinding boundaries. Gray‐dotted lines (⋯) represent the Monte Carlo simulation error interval.

**FIGURE A3:**
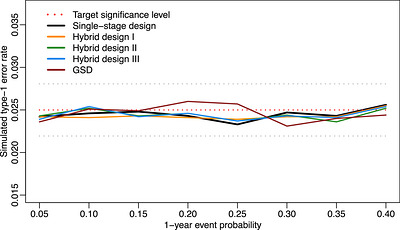
Simulated type‐1 error rates for n=360 (nominal level α=0.025, one‐sided). For hybrid design I: $\theta_{a}=0.5$ was used to derive the interim unblinding boundaries. Gray‐dotted lines (⋯) represent the Monte Carlo simulation error interval.

**FIGURE A4:**
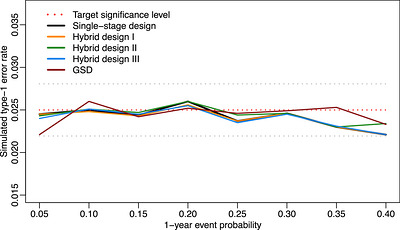
Simulated type‐1 error rates for n=720 (nominal level α=0.025, one‐sided). For hybrid design I: $\theta_{a}=0.6$ was used to derive the interim unblinding boundaries. Gray‐dotted lines (⋯) represent the Monte Carlo simulation error interval.

**FIGURE A5:**
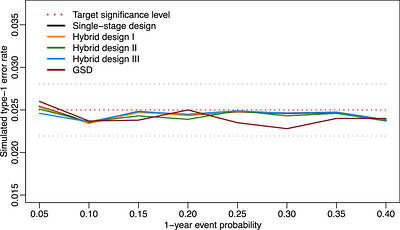
Simulated type‐1 error rates for n=1080 (nominal level α=0.025, one‐sided). For hybrid design I: $\theta_{a}=0.66$ was used to derive the interim unblinding boundaries. Gray‐dotted lines (⋯) represent the Monte Carlo simulation error interval.

**FIGURE A6:**
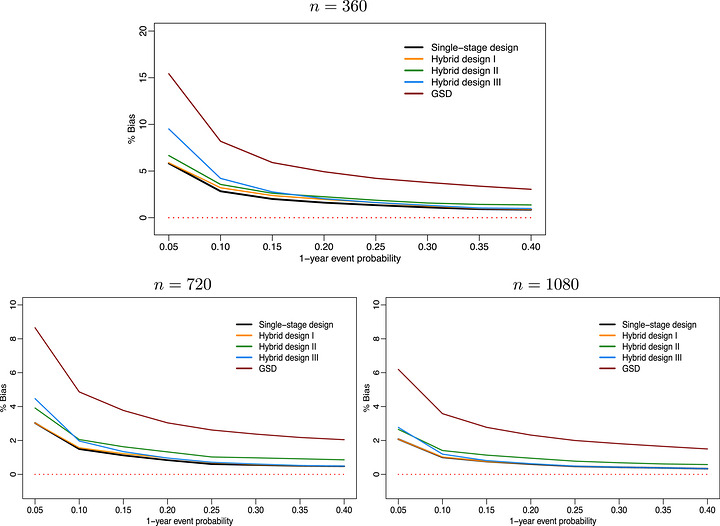
Bias of θ under $H_{0}:\theta =1$.

**FIGURE A7:**
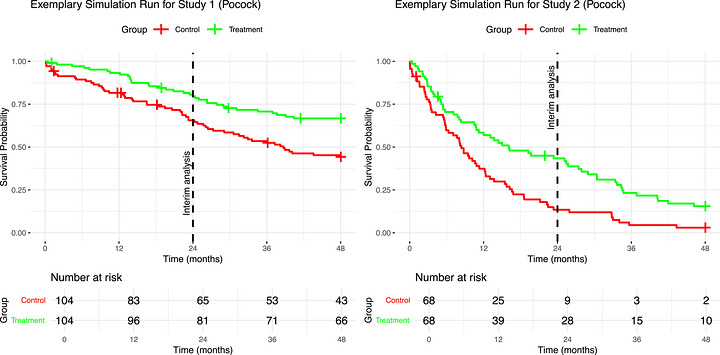
Exemplary Kaplan–Meier plots for the simulation settings considered in Section 3. In Study 1, the hazards $\lambda_{i}$ are low, so events occur infrequently, resulting in substantial administrative censoring at the end of the trial and relatively few events at the interim analysis. In Study 2, the hazards are higher, producing steeper Kaplan–Meier curves.

## Data Availability

The data that supports the findings of this study are available in the supplementary material of this article.

## Appendix A

### A.1 The Choice of $\Delta_{i}$

Figure A1 illustrates the upper and lower boundaries, $d_{l}$ and $d_{u}$, for all three hybrid designs discussed in Section 2.3, as a function of the unblinding parameter $\Delta_{i}$. It is important to note that the interpretation of $\Delta_{i}$ varies depending on the specific design. First, note that for relatively small values of $\Delta_{i}$, the unblinding boundaries are quite similar across the designs. In hybrid design I, the boundaries $d_{l}$ and $d_{u}$ depend on $\Delta_{1}$ in a nonlinear manner. Notably, $d_{u}$ increases rapidly as a function of $\Delta_{1}$. For certain values of $\Delta_{1}$, the mathematical expressions for $d_{l}$ and $d_{u}$ are undefined. In such cases, we set $d_{l}=0$ and $d_{u}$ to the expected sample size at interim, which is n=138 in this case. As a result, for some $\Delta_{1}$, the boundaries are configured such that unblinding becomes impossible, more concretely, if $\Phi \left(\sqrt{\frac{1}{4}D_{L}\log{\left(\theta_{a}\right)}^{2}}-\Phi^{-1}\left(1-\alpha_{1}\right)\right)\mp \Delta_{1}\notin \left(0,1\right)$. Hence, the admissible values of $\Delta_{1}$ depend on the specific study design, the sample size, and the assumed hazard ratio $\theta_{a}$.

Hybrid design II is based on quantile functions. Thus, there are some values of $\Delta_{i}$ where $d_{l}<d_{u}$ may occur. In these cases, we simply switch the boundaries. In our example, this adjustment happens at $\Delta_{2}=0.55$.

In hybrid design III, the boundaries $d_{l}$ and $d_{u}$ are linear functions of $\Delta_{i}$, as clearly illustrated in Figure A1

### A.2 Table of Estimands by Design

See Table A1.

**TABLE A1:** Details on type‐1 error rate, power, expected sample size, expected trial duration, and unblinding probability depending on the underlying study design.

Estimand	Single stage	Group sequential	Hybrid
Type‐1 error rate	$P_{H_{0}}\left(LR_{2}>c_{fix}\right)$	$P_{H_{0}}\left(LR_{1}>c_{1,e}\right)+P_{H_{0}}\left(LR_{1}\in \left[c_{f},c_{e,1}\right],LR_{1+2}>c_{e,2}\right)$	$P_{H_{0}}\left(LR_{1+2}>c_{fix}|d_{1}\in \left[d_{l},d_{u}\right]\right)+P_{H_{0}}\left(LR_{1}>c_{1,e}|d_{1}\notin \left[d_{l},d_{u}\right]\right)+P_{H_{0}}\left(LR_{1}\in \left[c_{f},c_{e,1}\right],LR_{1+2}>c_{e,2}|d_{1}\notin \left[d_{l},d_{u}\right]\right)$
Power	$P_{H_{1}}\left(LR_{2}>c_{fix}\right)$	$P_{H_{1}}\left(LR_{1}>c_{1,e}\right)+P_{H_{1}}\left(LR_{1}\in \left[c_{f},c_{e,1}\right],LR_{1+2}>c_{e,2}\right)$	$P_{H_{1}}\left(LR_{1+2}>c_{fix}|d_{1}\in \left[d_{l},d_{u}\right]\right)+P_{H_{1}}\left(LR_{1}>c_{1,e}|d_{1}\notin \left[d_{l},d_{u}\right]\right)+P_{H_{1}}\left(LR_{1}\in \left[c_{f},c_{e,1}\right],LR_{1+2}>c_{e,2}|d_{1}\notin \left[d_{l},d_{u}\right]\right)$
Expected sample size	$n_{1}+n_{2}$	$n_{1}+n_{2}P\left(LR_{1}\in \left[c_{f},c_{e,1}\right]\right)$	$n_{1}+n_{2}\left(P\left(LR_{1}\in \left[c_{f},c_{e,1}\right]|d_{1}\notin \left[d_{l},d_{u}\right]\right)+P\left(d_{1}\in \left[d_{l},d_{u}\right]\right)\right)$
Expected trial	$t_{final}$		
duration		$t_{interim}{+}^{*}t_{second\;stage}P\left(LR_{1}\in \left[c_{f},c_{e,1}\right]\right)$	$t_{interim}{+}^{*}t_{second\;stage}\left(P\left(LR_{1}\in \left[c_{f},c_{e,1}\right]|d_{1}\notin \left[d_{l},d_{u}\right]\right)+P\left(d_{1}\in \left[d_{l},d_{u}\right]\right)\right)$
Probability of unblinding	0	1	$P(d_{1}\notin \left[d_{l},d_{u}\right])$

### A.3 Type‐1 Error Rate Simulation

We add an additional short simulation study that investigates the type‐1 error rate of the proposed hybrid designs. Specifically, we consider a trial with a total target sample size of n∈{180,360,720,1080} participants, recruited over $t_{\text{rec}}=36$ months, resulting in an average recruitment rate of 5–30 patients per month. An interim analysis is planned after $t_{\text{interim}}=24$ months. We assume exponentially distributed survival times, with varying event probabilities $p_{A}=p_{B}$, yielding a constant hazard ratio of θ=1. The 1‐year censoring probabilities are set to 0.05 for all settings. Furthermore, we evaluate a group‐sequential design with Pocock boundaries as described in Section 2, using a one‐sided nominal significance level of α=0.025. It is important to note that the information fractions used for planning purposes—and consequently the derived designs and boundaries—vary depending on the assumptions about the 1‐year event probabilities. For the hybrid designs, we use the deviation parameters as presented in Section 2.3, resulting in different hybrid designs depending on the underlying assumed 1‐year $p_{B}$. Additionally, note that for the computation of the unblinding boundaries in hybrid design I, we selected $\theta_{a}$ such that the underlying equations are defined, and the boundaries approximately align with those of the other designs.

The simulated type‐1 error rates for the considered designs are summarized in Figures A2, A3, A4, A5, A6, A7, simulated based on 10,000 iterations. All figures include single‐stage designs, group‐sequential designs, and the three variants of the hybrid design under different assumptions for the 1‐year event probabilities.

Taking into account a Monte Carlo error of ±0.0031 (corresponding to the interval (0.0219,0.0281)), we conclude that the type‐1 error rate is adequately controlled across the various scenarios for all designs under consideration.

### A.7 Details on the Relationship Between the Unblinding Decision and the Decision of the Group‐Sequential Design

**TABLE A2:** Details on the relationship between the unblinding decision and the group‐sequential design decision in selected settings of Studies 1 and 2 using Pocock boundaries. All simulations were conducted with 10,000 iterations.

Study	Param.	Value	Method	$\hat{Corr}\left(LR_{1},d_{1}\right)$	n	n	$\hat{P}\left(GSD\;stops|Unblind\right)$	$\hat{P}\left(GSD\;continues|Blind\right)$
					unblinding	GSD stops		
1	$p_{B}$	0.05	Hybrid I	0.19	9981	2616	0.26	0.84
1	$p_{B}$	0.05	Hybrid II	0.19	9993	2616	0.26	0.71
1	$p_{B}$	0.05	Hybrid III	0.19	10000	2616	0.26	0.00
1	$p_{B}$	0.20	Hybrid I	0.15	666	3355	0.33	0.66
1	$p_{B}$	0.20	Hybrid II	0.15	1351	3355	0.34	0.66
1	$p_{B}$	0.20	Hybrid III	0.15	2566	3355	0.33	0.66
1	$p_{B}$	0.60	Hybrid I	0.10	10000	7652	0.77	0.00
1	$p_{B}$	0.60	Hybrid II	0.10	10000	7652	0.77	0.00
1	$p_{B}$	0.60	Hybrid III	0.10	10000	7652	0.77	0.00
1	θ	0.20	Hybrid I	0.37	1677	7601	0.59	0.21
1	θ	0.20	Hybrid II	0.37	2514	7601	0.60	0.18
1	θ	0.20	Hybrid III	0.37	4460	7601	0.67	0.17
1	θ	0.47	Hybrid I	0.15	666	3353	0.33	0.66
1	θ	0.47	Hybrid II	0.15	1349	3353	0.33	0.66
1	θ	0.47	Hybrid III	0.15	2566	3353	0.33	0.66
1	θ	1.00	Hybrid I	−0.01	3474	5153	0.52	0.49
1	θ	1.00	Hybrid II	−0.01	4982	5153	0.51	0.48
1	θ	1.00	Hybrid III	−0.01	5812	5153	0.51	0.48
2	$p_{B}$	0.10	Hybrid I	0.15	10000	2867	0.29	0.00
2	$p_{B}$	0.10	Hybrid II	0.15	10000	2867	0.29	0.00
2	$p_{B}$	0.10	Hybrid III	0.15	10000	2867	0.29	0.00
2	$p_{B}$	0.60	Hybrid I	0.08	420	4204	0.39	0.58
2	$p_{B}$	0.60	Hybrid II	0.08	1984	4204	0.41	0.58
2	$p_{B}$	0.60	Hybrid III	0.08	1330	4204	0.39	0.58
2	$p_{B}$	0.80	Hybrid I	0.06	4896	5422	0.57	0.48
2	$p_{B}$	0.80	Hybrid II	0.06	8284	5422	0.55	0.50
2	$p_{B}$	0.80	Hybrid III	0.06	6983	5422	0.56	0.49
2	θ	0.20	Hybrid I	0.27	3544	9838	0.97	0.01
2	θ	0.20	Hybrid II	0.27	5758	9838	0.98	0.01
2	θ	0.20	Hybrid III	0.27	5749	9838	0.98	0.01
2	θ	0.56	Hybrid I	0.08	420	4203	0.39	0.58
2	θ	0.56	Hybrid II	0.08	1983	4203	0.41	0.58
2	θ	0.56	Hybrid III	0.08	1327	4203	0.39	0.58
2	θ	1.00	Hybrid I	0.00	1671	5089	0.50	0.49
2	θ	1.00	Hybrid II	0.00	5196	5089	0.50	0.48
2	θ	1.00	Hybrid III	0.00	3548	5089	0.50	0.49
