# Supplementary material for: Blinded‐Into‐Unblinded Interim Analyses for Clinical Trials With Time‐to‐Event Endpoints
Source: Biom J. 2026 Jul 24;68(4):e70151. doi: 10.1002/bimj.70151 (PMC13397411; doi:10.1002/bimj.70151)
Supplement: Supplementary file 1 — Supporting File 1: bimj70151‐sup‐0001‐SuppMat.pdf. [file BIMJ-68-e70151-s002.pdf]

Biometrical Journal **XX** (20XX) XX, zzz–zzz / DOI: 10.1002/bimj.200100000

Supplementary Material for: Blinded-into-unblinded interim analyses for clinical trials with time-to-event endpoints

Stephen Schüürhuis [1], **Jan Meis** [2], **Björn Bokelmann** [1], **Meinhard Kieser** [2], and **Carolin Herrmann**[*, 3]

[1] Institute of Biometry and Clinical Epidemiology, Charité - Universitätsmedizin Berlin, Corporate member of Freie Universität Berlin and Humboldt-Universität zu Berlin, Charitéplatz 1, 10117 Berlin, Germany

[2] Institute of Medical Biometry, University of Heidelberg, Im Neuenheimer Feld 130.3, 69120 Heidelberg, Germany

[3] Mathematical Institute, Heinrich Heine University Düsseldorf, Universitätsstraße 1, 40225 Düsseldorf, Germany

*Corresponding author: carolin.herrmann@hhu.de

© 20XX WILEY-VCH Verlag GmbH & Co. KGaA, Weinheim

Supplement 1: Plots of performance characteristics in Study 1 and 2 when planning the trial with O'Brien-Fleming boundaries

In addition to using the Pocock boundaries, we also conducted the simulation study with O'Brien–Fleming boundaries to examine performance characteristics under less liberal interim efficacy boundaries. The table below presents the planning parameters, which are identical to those used in the Pocock case, along with the resulting design. Note that only the required sample size as well the as expected number of events differ.

Table 1 Parameter values for two-stage group-sequential reference study designs for a study with $t_{\text{rec}} = 36$ and $t_{\text{interim}} = 24$. Note that the information I_1 is approximate and the expected number of events rounded to the closest integer.

**Parameter**	**Study 1**	**Study 2**
Hazard ratio $\theta_a = h_V/h_P$	0.472	0.557
One-sided type-1 error rate	0.025	0.025
Interim information I_1	0.3	0.4
Power	0.8	0.8
Type of design	O'Brien-Fleming	O'Brien-Fleming
Binding futility boundary c_f	$c_f = 0$	$c_f = 0$
Required sample size n	184	122
Expected number of events at interim under $\mathcal{H}_1$	20	41
Expected number of events at final under $\mathcal{H}_1$	66	105

For Hybrid Designs II and III, we used the same parameters as in the main paper. For Hybrid Design I, however, we chose to trigger unblinding if the interim power deviated by $\pm 5\%$, rather than the $\pm 10\%$ used in the Pocock case. This stricter threshold was selected because, under O'Brien–Fleming boundaries, the interim power is already relatively low due to the stringent stopping criterion for efficacy. This choice resulted in the following unblinding boundaries:

- Hybrid design I: $\{0; 32\}$ in Study 1 and $\{22; 49\}$ in Study 2,
- Hybrid design II: $\{12; 23\}$ in Study 1 and $\{30; 44\}$ in Study 2,
- Hybrid design III: $\{14; 22\}$ in Study 1 and $\{29; 45\}$ in Study 2.

The performance characteristics can be seen in the following Figures 1-4.

© 20XX WILEY-VCH Verlag GmbH & Co. KGaA, Weinheim

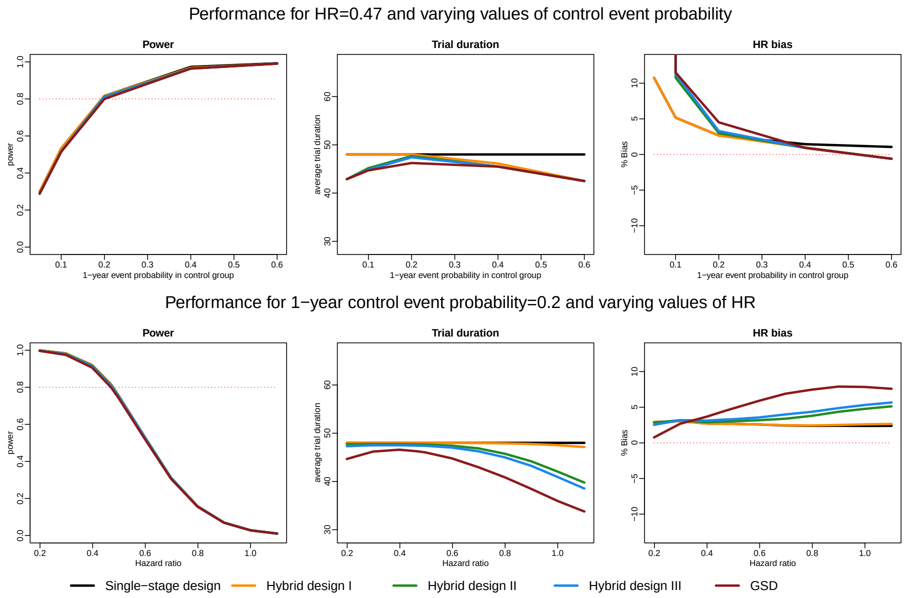

Figure 1 Power, expected trial duration and relative bias of the hazard ratio in Study 1 using O'Brien-Fleming boundaries.

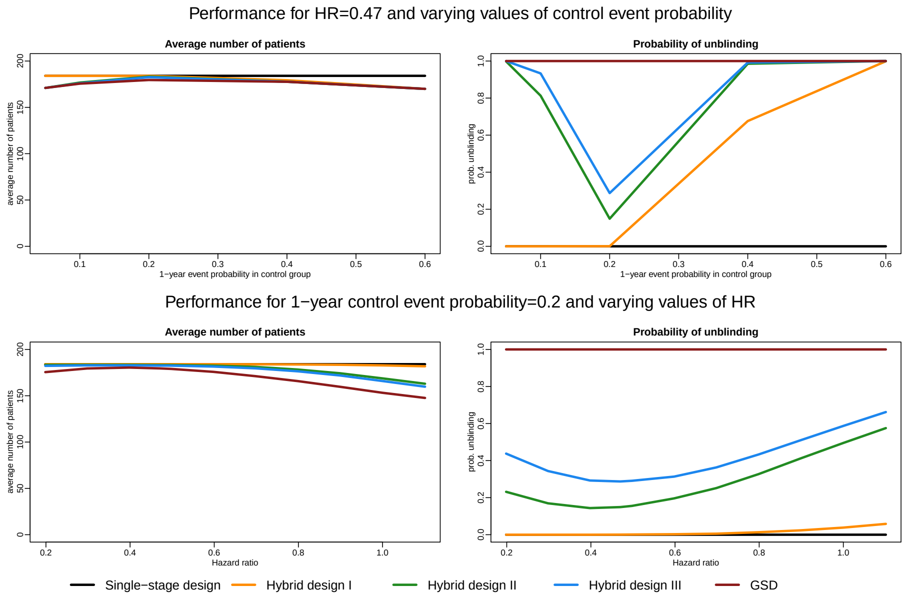

Figure 2 Expected sample size and probability of unblinding in Study 1 using O'Brien-Fleming boundaries.

© 20XX WILEY-VCH Verlag GmbH & Co. KGaA, Weinheim

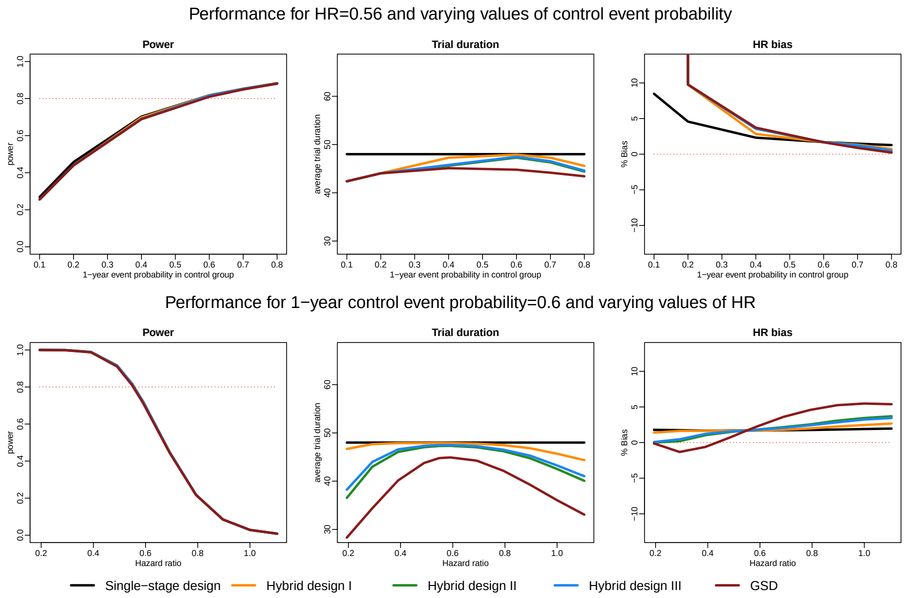

Figure 3 Power, expected trial duration and relative bias of the hazard ratio in Study 2 using O'Brien-Fleming boundaries.

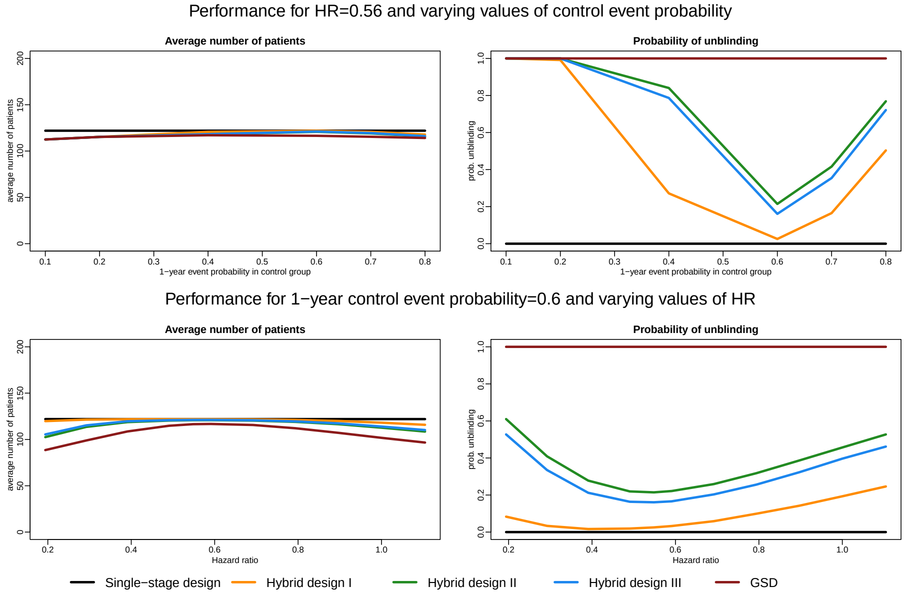

Figure 4 Expected sample size and probability of unblinding in Study 2 using O'Brien-Fleming boundaries.

© 20XX WILEY-VCH Verlag GmbH & Co. KGaA, Weinheim
