# Supplementary material for: Blinded‐Into‐Unblinded Interim Analyses for Clinical Trials With Time‐to‐Event Endpoints
Source: Biom J. 2026 Jul 24;68(4):e70151. doi: 10.1002/bimj.70151 (PMC13397411; doi:10.1002/bimj.70151)
Supplement: Supplementary file 2 — Supporting File 2: bimj70151‐sup‐0002‐Datacode.zip. [file BIMJ-68-e70151-s001.zip › R_Code_Submission_wo_data/Appendix/Figure10.pdf]

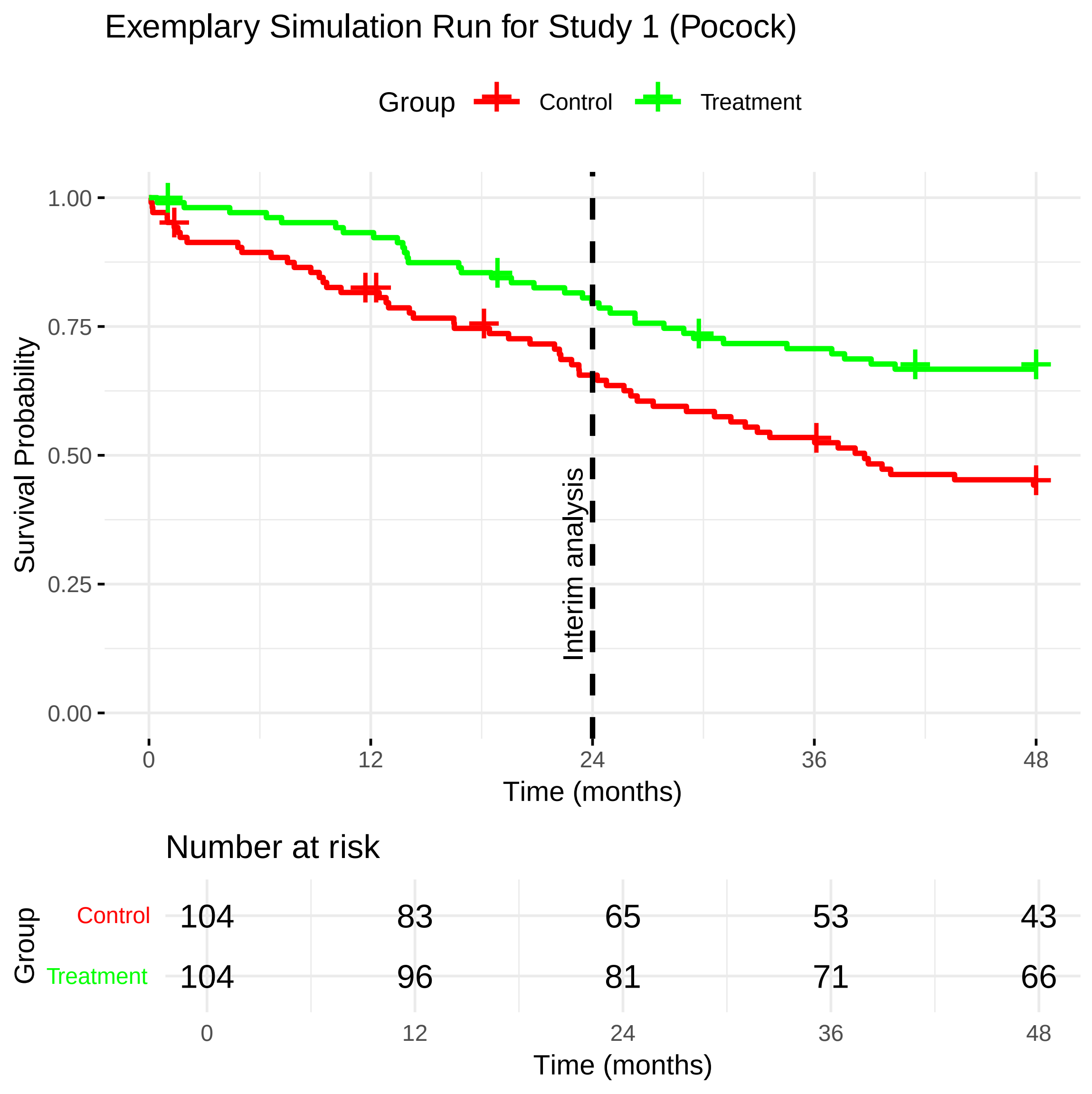
Exemplary Simulation Run for Study 1 (Pocock)
Group
Control
Treatment
Survival Probability
1.00
0.75
0.50
0.25
0.00
Interim analysis
0
12
24
36
48
Time (months)
Number at risk
Group
Control 104 83 65 53 43
Treatment 104 96 81 71 66
0
12
24
36
48
Time (months)

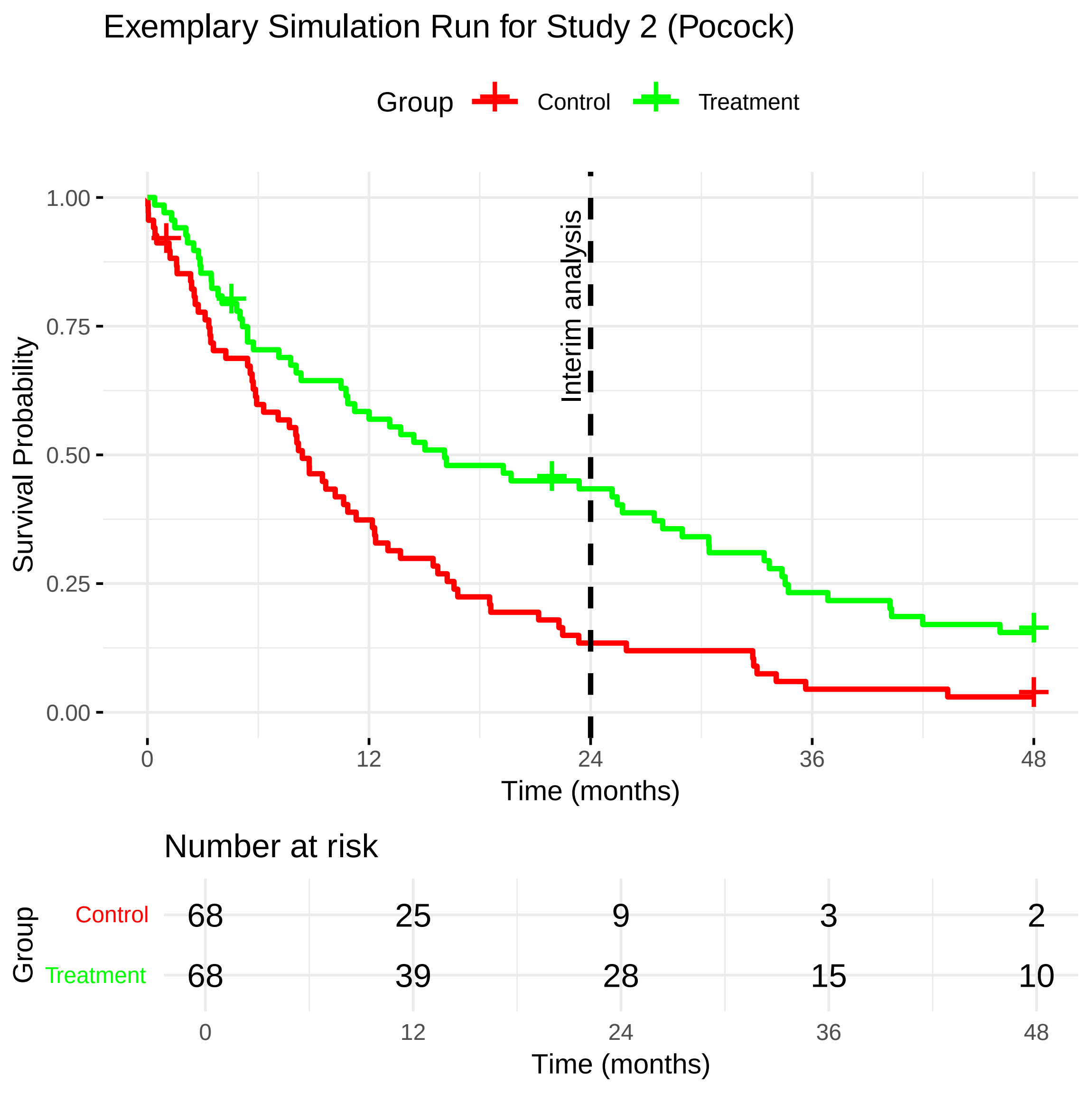
Exemplary Simulation Run for Study 2 (Pocock)
Group
Control
Treatment
Survival Probability
1.00
0.75
0.50
0.25
0.00
Interim analysis
0
12
24
36
48
Time (months)
Number at risk
Group
Control 68 25 9 3 2
Treatment 68 39 28 15 10
0
12
24
36
48
Time (months)
