# Supplementary material for: Blinded‐Into‐Unblinded Interim Analyses for Clinical Trials With Time‐to‐Event Endpoints
Source: Biom J. 2026 Jul 24;68(4):e70151. doi: 10.1002/bimj.70151 (PMC13397411; doi:10.1002/bimj.70151)
Supplement: Supplementary file 2 — Supporting File 2: bimj70151‐sup‐0002‐Datacode.zip. [file BIMJ-68-e70151-s001.zip › R_Code_Submission_wo_data/Appendix/Figure4.pdf]

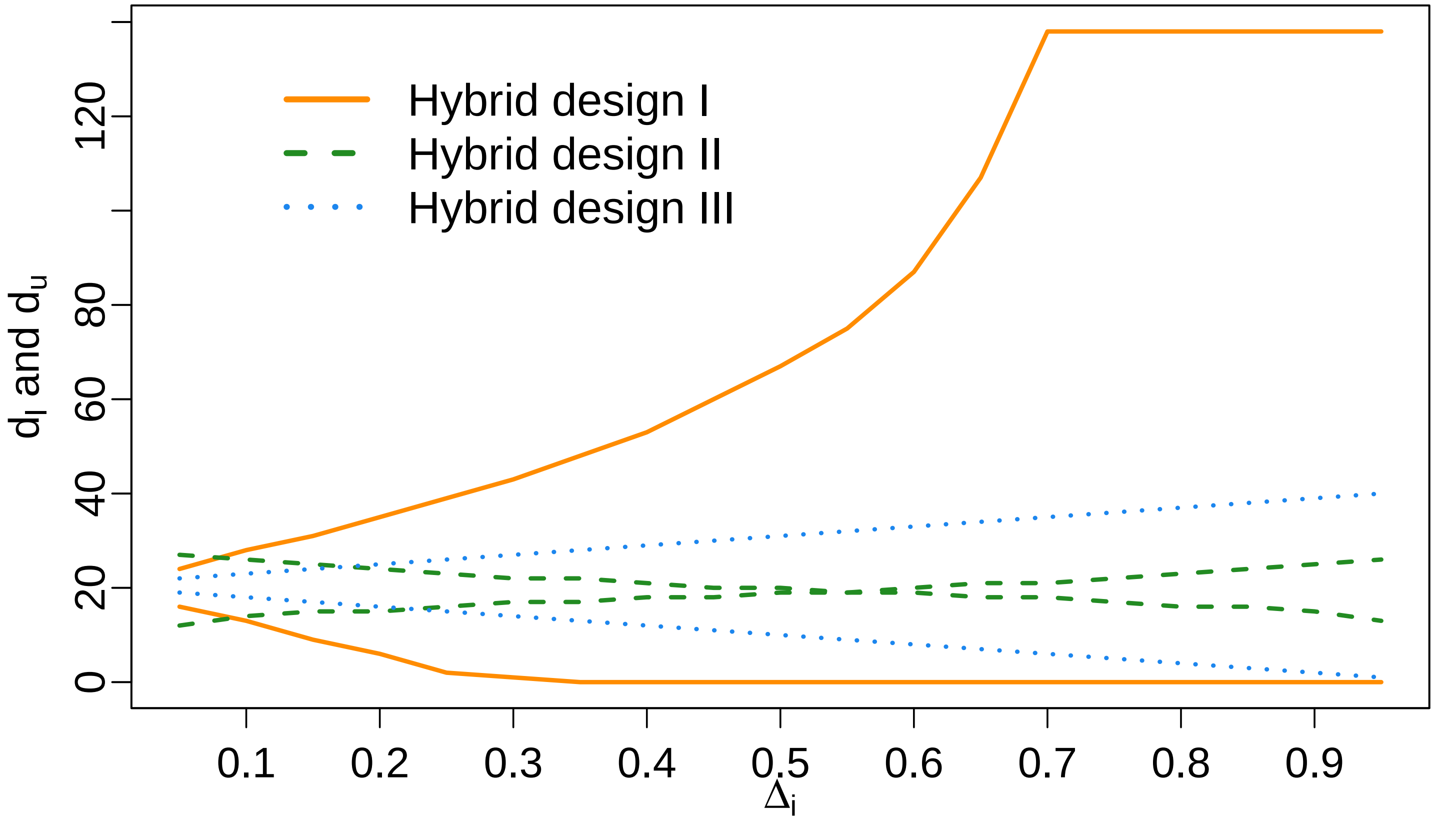

Hybrid design I
Hybrid design II
Hybrid design III
0
20
40
60
80
120
0.1
0.2
0.3
0.4
0.5
0.6
0.7
0.8
0.9
d_l and d_u
Δ_i
