# Supplementary material for: Blinded‐Into‐Unblinded Interim Analyses for Clinical Trials With Time‐to‐Event Endpoints
Source: Biom J. 2026 Jul 24;68(4):e70151. doi: 10.1002/bimj.70151 (PMC13397411; doi:10.1002/bimj.70151)
Supplement: Supplementary file 2 — Supporting File 2: bimj70151‐sup‐0002‐Datacode.zip. [file BIMJ-68-e70151-s001.zip › R_Code_Submission_wo_data/README.html]

Code Documentation


## Contents

- 1 Content
- 2 File and Folder-Structure
- 3 How to reproduce the results shown in the paper
- 4 Helper scripts
- 5 Working with intermediate results
- 6 Session Info

# Code Documentation

 Code

- Show All Code
- Hide All Code
- ---
- View Source

Blinded-into-unblinded interim analyses for clinical trials with time-to-event endpoints

Author

Stephen Schueuerhuis

## 1 Content

This files contains a short documentation of the code belonging to the submitted paper ***Blinded-into-unblinded interim analyses for clinical trials with time-to-event endpoints*** and illustrates how the results shown in the paper may be reproduced.

To begin working with the submitted code, open the R project file `blinded_into_unblinded.Rproj`. This ensures that all file paths are correctly set relative to the project root.

## 2 File and Folder-Structure

The project contains the following files:

File-Structure

| Name | Description |
| --- | --- |
| `0_util.R` | Contains helper functions for data simulation, computation of test statistics, and translation of survival parameters. |
| `1_data_simulation.R` | Simulates the data for all considered simulation settings and saves them in *Simulation Study/Data*. |
| `2_design_creation.R` | Creates study designs (`create_hybrid_design())`for all considered simulation settings and saves them in *Simulation Study/Designs*. |
| `3_evaluation.R` | Imports the data from *Simulation Study/Data* and the designs from *Simulation Study/Designs*. Computes performance characteristics for all designs across all data settings and saves the results as raw data and in aggregated form in *Simulation Study/Results*. |
| `4_plots.R` | Imports the results from *Simulation Study/Results*, generates the plots shown in the paper, and saves them in *Simulation Study/Plots* |
| `example_S.5.R` | Reproduces the example from Section 5 of the paper. The corresponding data, design, and results are saved in the *Example* folder. |
| `appendix_A.1.R` | Reproduces the plot shown in Appendix A.1 of the paper and saves it in the *Appendix* folder. |
| `appendix_A.5.R` | Reproduces the plot shown in Appendix A.5 of the paper and saves it in the *Appendix* folder. |
| `appendix_A.7.R` | Reproduces the table shown in Appendix A.7 of the paper and saves it as a *.csv* file in the *Appendix* folder. |
| `master_script.R` | Master script that sources the files  `1_data_simulation.R, 2_design_creation.R, 3_evaluation.R`, and `4_plots.R` in sequence to reproduce the entire simulation study. It also sources `example_S.5.R`, `appendix_A.1.R`, `appendix_A.5.R`, and `appendix_A.7.R` to reproduce the example and the appendix figures/tables.  **Note:** Reproducing the full simulation study is computationally intensive. |
| `repro_example.R` | To potentially prevent having to rerun the whole simulation study, we provide the script `repro_example.R.` This script enables reproducing Figures 2 and 3 from the paper, corresponding to the simulation Study 1 using Pocock-Boundaries in the paper. The corresponding data, designs, results, and plots are saved in the *Reproduction Rexample* folder. |

The project contains the following folders:

File-Structure

| Name | Description |
| --- | --- |
| *Simulation Study* | The *Simulation Study* folder contains all data, designs, results, and plots for the considered simulation settings. Its structure is as follows:  *Simulation Study*   - *Data* - *Designs* - *Results* - Plots   Each of the four main folders (*Data, Designs, Results, Plots*) contains the following subfolders:   - *Performance*    - *Study\_1\_Pocock*   - *Study\_2\_Pocock*   - *Study\_1\_O’Brien-Fleming*   - *Study\_2\_O’Brien-Fleming* - *Type-1 Error Rate and Bias*    - *n=180*   - *n=360*   - *n=720*   - n=1080   That is, the *Performance* folder includes the complete simulation study for all performance characteristics:   - **Pocock** design: Figures 2, 3, 11, and 12 in the main paper. - **O’Brien-Fleming** design: Figures 1–4 in the Supplement.   Moreover, the *Type-1 Error Rate* folder contains the simulation results for bias and type-1 error rate across all sample sizes, corresponding to Appendix A.3 and A.4 (Figures 5–9). |
| *Reproduction Example* | The file `master_script.R` reruns the whole simulation study by sequentially calling the corresponding script-files. Due to the long runtime of the whole simulation study, we provide the script `repro_example.R` that reproduces the results for simulation study 1, using Pocock-boundaries. The data, designs, results and plots are saved in the folder *Reproduction Example.* |
| *Example* | This folder contains the data, designs, and results for the example discussed in Section 5 of the main paper. Structurally, this script is equivalent to the simulation scripts, with the key difference that it generates a single dataset, which is then analyzed using all designs. |
| *Appendix* | This folder contains the output created by the R-files `appendix_A.1.R`, `appendix_A.5.R` and `appendix_A.7.R`. |

## 3 How to reproduce the results shown in the paper

To reproduce the results in the paper, the file `master_script.R` needs to be run. All output (data, designs, results, plots) will automatically be saved in the corresponding folder, mainly in the *Simulation Study* folder. Plots-files are named according to their appearance in the paper. Reproducibility is ensured by using seeds set in the script `1_data_simulation.R` prior to the data simulation in all simulation settings.

Due to the long runtime of the whole simulation study, we provide the script `repro_example.R` that reprocudes the results for simulation study 1, using Pocock-boundaries. The data, designs, results and plots are saved in the folder *Reproduction Example.* Hence, running this script reproduces Figure 2 and Figure 3 shown in the paper.

The results presented in Section 5 (Study Example) and in the Appendix (A.1, A.5, and A.7) can be reproduced by running the corresponding R scripts, as outlined above.

## 4 Helper scripts

Helper functions are contained in the file `0_util.R`. The file is organized into the following sections:

- **Trial simulation:** Functions for simulating study entry times, survival outcomes, and censoring data for treatment and control groups under specified distributional assumptions. It also includes a function that generates a dataset at a given interim timepoint.
- **Test statistics:** A wrapper function around `survdiff()` that extracts the normally distributed version of the log-rank test statistic from the *survival* package. Additionally, a function for computing the inverse normal test statistic is provided. Note, however, that in the paper’s Methods section, we use the cumulative test statistic for testing.
- **Survival parameters:** Functions for translating between different parameterizations of survival models—such as event probabilities, hazards, and median survival times—under the assumption of an exponential distribution.
- **Study design:** A wrapper function that constructs a study design, including its boundary set, and defines unblinding boundaries for the hybrid designs.
- **Event estimation:** Functions for estimating the expected number of events at a given time, based on assumptions about survival and censoring. These functions are used to compute the boundaries of the hybrid designs, as described in Sections 2.2.4–2.2.6 of the paper.

For details, we refer to the comments in the corresponding code file.

## 5 Working with intermediate results

The data files are large (several GB) and therefore not included with the code. However, all result files generated by `3_evaluation.R` have been uploaded to the corresponding results folder, as outlined above. Consequently, running `4_plots.R` will reproduce all plots shown in the paper without the need to re-run the full data simulation.

## 6 Session Info

The example results presented in this paper that were run on a local computer using the following R packages and their corresponding versions:

Code

```
sessionInfo()
```

```
R version 4.5.1 (2025-06-13 ucrt)
Platform: x86_64-w64-mingw32/x64
Running under: Windows 10 x64 (build 19045)

Matrix products: default
  LAPACK version 3.12.1

locale:
[1] LC_COLLATE=German_Germany.utf8  LC_CTYPE=German_Germany.utf8   
[3] LC_MONETARY=German_Germany.utf8 LC_NUMERIC=C                   
[5] LC_TIME=German_Germany.utf8    

time zone: Europe/Berlin
tzcode source: internal

attached base packages:
[1] stats     graphics  grDevices utils     datasets  methods   base     

other attached packages:
 [1] survminer_0.5.0   ggpubr_0.6.1      ggplot2_3.5.2     dplyr_1.1.4      
 [5] plyr_1.8.9        tidyr_1.3.1       rpact_4.2.1       survival_3.8-3   
 [9] magrittr_2.0.3    data.table_1.17.8

loaded via a namespace (and not attached):
 [1] rappdirs_0.3.3     generics_0.1.4     rstatix_0.7.2      lattice_0.22-7    
 [5] digest_0.6.37      evaluate_1.0.4     grid_4.5.1         RColorBrewer_1.1-3
 [9] fastmap_1.2.0      jsonlite_2.0.0     Matrix_1.7-3       backports_1.5.0   
[13] Formula_1.2-5      gridExtra_2.3      purrr_1.1.0        scales_1.4.0      
[17] abind_1.4-8        cli_3.6.5          KMsurv_0.1-6       rlang_1.1.6       
[21] splines_4.5.1      withr_3.0.2        yaml_2.3.10        tools_4.5.1       
[25] ggsignif_0.6.4     km.ci_0.5-6        broom_1.0.9        vctrs_0.6.5       
[29] R6_2.6.1           zoo_1.8-14         lifecycle_1.0.4    car_3.1-3         
[33] pkgconfig_2.0.3    pillar_1.11.0      gtable_0.3.6       glue_1.8.0        
[37] Rcpp_1.1.0         xfun_0.52          tibble_3.3.0       tidyselect_1.2.1  
[41] rstudioapi_0.17.1  knitr_1.50         xtable_1.8-4       farver_2.1.2      
[45] survMisc_0.5.6     htmltools_0.5.8.1  rmarkdown_2.29     carData_3.0-5     
[49] compiler_4.5.1
```


##### Source Code

```
---
title: "Code Documentation"
subtitle: "Blinded-into-unblinded interim analyses for clinical trials with time-to-event endpoints"
author: 
  - name: "Stephen Schueuerhuis"
format:
  html:
    toc: true
    toc-depth: 3
    toc-location: right
    toc-title: "Contents"
    number-sections: true
    smooth-scroll: true
    theme: cosmo  # You can change to flatly, journal, simplex, etc.
    code-fold: true
    code-tools: true
    highlight-style: github
editor: visual
---

```{r}
#| echo: false
#| results: false
#| message: false
#| warning: false


# To show all relevant packages in the session info
source("0_util.R")
```

## Content

This files contains a short documentation of the code belonging to the submitted paper ***Blinded-into-unblinded interim analyses for clinical trials with time-to-event endpoints*** and illustrates how the results shown in the paper may be reproduced.

To begin working with the submitted code, open the R project file `blinded_into_unblinded.Rproj`. This ensures that all file paths are correctly set relative to the project root.

## File and Folder-Structure

The project contains the following files:

+-----------------------+----------------------------------------------------------------------------------------------------------------------------------------------------------------------------------------------------------------------------------------------------------------------------------------------------------------------------------------------------------------+
| Name                  | Description                                                                                                                                                                                                                                                                                                                                                    |
+=======================+================================================================================================================================================================================================================================================================================================================================================================+
| `0_util.R`            | Contains helper functions for data simulation, computation of test statistics, and translation of survival parameters.                                                                                                                                                                                                                                         |
+-----------------------+----------------------------------------------------------------------------------------------------------------------------------------------------------------------------------------------------------------------------------------------------------------------------------------------------------------------------------------------------------------+
| `1_data_simulation.R` | Simulates the data for all considered simulation settings and saves them in *Simulation Study/Data*.                                                                                                                                                                                                                                                           |
+-----------------------+----------------------------------------------------------------------------------------------------------------------------------------------------------------------------------------------------------------------------------------------------------------------------------------------------------------------------------------------------------------+
| `2_design_creation.R` | Creates study designs (`create_hybrid_design())`for all considered simulation settings and saves them in *Simulation Study/Designs*.                                                                                                                                                                                                                           |
+-----------------------+----------------------------------------------------------------------------------------------------------------------------------------------------------------------------------------------------------------------------------------------------------------------------------------------------------------------------------------------------------------+
| `3_evaluation.R`      | Imports the data from *Simulation Study/Data* and the designs from *Simulation Study/Designs*. Computes performance characteristics for all designs across all data settings and saves the results as raw data and in aggregated form in *Simulation Study/Results*.                                                                                           |
+-----------------------+----------------------------------------------------------------------------------------------------------------------------------------------------------------------------------------------------------------------------------------------------------------------------------------------------------------------------------------------------------------+
| `4_plots.R`           | Imports the results from *Simulation Study/Results*, generates the plots shown in the paper, and saves them in *Simulation Study/Plots*                                                                                                                                                                                                                        |
+-----------------------+----------------------------------------------------------------------------------------------------------------------------------------------------------------------------------------------------------------------------------------------------------------------------------------------------------------------------------------------------------------+
| `example_S.5.R`       | Reproduces the example from Section 5 of the paper. The corresponding data, design, and results are saved in the *Example* folder.                                                                                                                                                                                                                             |
+-----------------------+----------------------------------------------------------------------------------------------------------------------------------------------------------------------------------------------------------------------------------------------------------------------------------------------------------------------------------------------------------------+
| `appendix_A.1.R`      | Reproduces the plot shown in Appendix A.1 of the paper and saves it in the *Appendix* folder.                                                                                                                                                                                                                                                                  |
+-----------------------+----------------------------------------------------------------------------------------------------------------------------------------------------------------------------------------------------------------------------------------------------------------------------------------------------------------------------------------------------------------+
| `appendix_A.5.R`      | Reproduces the plot shown in Appendix A.5 of the paper and saves it in the *Appendix* folder.                                                                                                                                                                                                                                                                  |
+-----------------------+----------------------------------------------------------------------------------------------------------------------------------------------------------------------------------------------------------------------------------------------------------------------------------------------------------------------------------------------------------------+
| `appendix_A.7.R`      | Reproduces the table shown in Appendix A.7 of the paper and saves it as a *.csv* file in the *Appendix* folder.                                                                                                                                                                                                                                                |
+-----------------------+----------------------------------------------------------------------------------------------------------------------------------------------------------------------------------------------------------------------------------------------------------------------------------------------------------------------------------------------------------------+
| `master_script.R`     | Master script that sources the files\                                                                                                                                                                                                                                                                                                                          |
|                       | `1_data_simulation.R, 2_design_creation.R, 3_evaluation.R`, and `4_plots.R` in sequence to reproduce the entire simulation study. It also sources `example_S.5.R`, `appendix_A.1.R`, `appendix_A.5.R`, and `appendix_A.7.R` to reproduce the example and the appendix figures/tables.                                                                          |
|                       |                                                                                                                                                                                                                                                                                                                                                                |
|                       | **Note:** Reproducing the full simulation study is computationally intensive.                                                                                                                                                                                                                                                                                  |
+-----------------------+----------------------------------------------------------------------------------------------------------------------------------------------------------------------------------------------------------------------------------------------------------------------------------------------------------------------------------------------------------------+
| `repro_example.R`     | To potentially prevent having to rerun the whole simulation study, we provide the script `repro_example.R.` This script enables reproducing Figures 2 and 3 from the paper, corresponding to the simulation Study 1 using Pocock-Boundaries in the paper. The corresponding data, designs, results, and plots are saved in the *Reproduction Rexample* folder. |
+-----------------------+----------------------------------------------------------------------------------------------------------------------------------------------------------------------------------------------------------------------------------------------------------------------------------------------------------------------------------------------------------------+

: File-Structure {tbl-colwidths="\[20,80\]"}

The project contains the following folders:

+------------------------+---------------------------------------------------------------------------------------------------------------------------------------------------------------------------------------------------------------------------------------------------------------------------------------------------------------------------------------------------------------------------------------+
| Name                   | Description                                                                                                                                                                                                                                                                                                                                                                           |
+========================+=======================================================================================================================================================================================================================================================================================================================================================================================+
| *Simulation Study*     | The *Simulation Study* folder contains all data, designs, results, and plots for the considered simulation settings. Its structure is as follows:                                                                                                                                                                                                                                     |
|                        |                                                                                                                                                                                                                                                                                                                                                                                       |
|                        | *Simulation Study*                                                                                                                                                                                                                                                                                                                                                                    |
|                        |                                                                                                                                                                                                                                                                                                                                                                                       |
|                        | -   *Data*                                                                                                                                                                                                                                                                                                                                                                            |
|                        |                                                                                                                                                                                                                                                                                                                                                                                       |
|                        | -   *Designs*                                                                                                                                                                                                                                                                                                                                                                         |
|                        |                                                                                                                                                                                                                                                                                                                                                                                       |
|                        | -   *Results*                                                                                                                                                                                                                                                                                                                                                                         |
|                        |                                                                                                                                                                                                                                                                                                                                                                                       |
|                        | -   Plots                                                                                                                                                                                                                                                                                                                                                                             |
|                        |                                                                                                                                                                                                                                                                                                                                                                                       |
|                        | Each of the four main folders (*Data, Designs, Results, Plots*) contains the following subfolders:                                                                                                                                                                                                                                                                                    |
|                        |                                                                                                                                                                                                                                                                                                                                                                                       |
|                        | -   *Performance*                                                                                                                                                                                                                                                                                                                                                                     |
|                        |                                                                                                                                                                                                                                                                                                                                                                                       |
|                        |     -   *Study_1_Pocock*                                                                                                                                                                                                                                                                                                                                                              |
|                        |                                                                                                                                                                                                                                                                                                                                                                                       |
|                        |     -   *Study_2_Pocock*                                                                                                                                                                                                                                                                                                                                                              |
|                        |                                                                                                                                                                                                                                                                                                                                                                                       |
|                        |     -   *Study_1_O'Brien-Fleming*                                                                                                                                                                                                                                                                                                                                                     |
|                        |                                                                                                                                                                                                                                                                                                                                                                                       |
|                        |     -   *Study_2_O'Brien-Fleming*                                                                                                                                                                                                                                                                                                                                                     |
|                        |                                                                                                                                                                                                                                                                                                                                                                                       |
|                        | -   *Type-1 Error Rate and Bias*                                                                                                                                                                                                                                                                                                                                                      |
|                        |                                                                                                                                                                                                                                                                                                                                                                                       |
|                        |     -   *n=180*                                                                                                                                                                                                                                                                                                                                                                       |
|                        |                                                                                                                                                                                                                                                                                                                                                                                       |
|                        |     -   *n=360*                                                                                                                                                                                                                                                                                                                                                                       |
|                        |                                                                                                                                                                                                                                                                                                                                                                                       |
|                        |     -   *n=720*                                                                                                                                                                                                                                                                                                                                                                       |
|                        |                                                                                                                                                                                                                                                                                                                                                                                       |
|                        |     -   n=1080                                                                                                                                                                                                                                                                                                                                                                        |
|                        |                                                                                                                                                                                                                                                                                                                                                                                       |
|                        | That is, the *Performance* folder includes the complete simulation study for all performance characteristics:                                                                                                                                                                                                                                                                         |
|                        |                                                                                                                                                                                                                                                                                                                                                                                       |
|                        | -   **Pocock** design: Figures 2, 3, 11, and 12 in the main paper.                                                                                                                                                                                                                                                                                                                    |
|                        |                                                                                                                                                                                                                                                                                                                                                                                       |
|                        | -   **O’Brien-Fleming** design: Figures 1–4 in the Supplement.                                                                                                                                                                                                                                                                                                                        |
|                        |                                                                                                                                                                                                                                                                                                                                                                                       |
|                        | Moreover, the *Type-1 Error Rate* folder contains the simulation results for bias and type-1 error rate across all sample sizes, corresponding to Appendix A.3 and A.4 (Figures 5–9).                                                                                                                                                                                                 |
+------------------------+---------------------------------------------------------------------------------------------------------------------------------------------------------------------------------------------------------------------------------------------------------------------------------------------------------------------------------------------------------------------------------------+
| *Reproduction Example* | The file `master_script.R` reruns the whole simulation study by sequentially calling the corresponding script-files. Due to the long runtime of the whole simulation study, we provide the script `repro_example.R` that reproduces the results for simulation study 1, using Pocock-boundaries. The data, designs, results and plots are saved in the folder *Reproduction Example.* |
+------------------------+---------------------------------------------------------------------------------------------------------------------------------------------------------------------------------------------------------------------------------------------------------------------------------------------------------------------------------------------------------------------------------------+
| *Example*              | This folder contains the data, designs, and results for the example discussed in Section 5 of the main paper. Structurally, this script is equivalent to the simulation scripts, with the key difference that it generates a single dataset, which is then analyzed using all designs.                                                                                                |
+------------------------+---------------------------------------------------------------------------------------------------------------------------------------------------------------------------------------------------------------------------------------------------------------------------------------------------------------------------------------------------------------------------------------+
| *Appendix*             | This folder contains the output created by the R-files `appendix_A.1.R`, `appendix_A.5.R` and `appendix_A.7.R`.                                                                                                                                                                                                                                                                       |
+------------------------+---------------------------------------------------------------------------------------------------------------------------------------------------------------------------------------------------------------------------------------------------------------------------------------------------------------------------------------------------------------------------------------+

: File-Structure {tbl-colwidths="\[20,80\]"}

## How to reproduce the results shown in the paper

To reproduce the results in the paper, the file `master_script.R` needs to be run. All output (data, designs, results, plots) will automatically be saved in the corresponding folder, mainly in the *Simulation Study* folder. Plots-files are named according to their appearance in the paper. Reproducibility is ensured by using seeds set in the script `1_data_simulation.R` prior to the data simulation in all simulation settings.

Due to the long runtime of the whole simulation study, we provide the script `repro_example.R` that reprocudes the results for simulation study 1, using Pocock-boundaries. The data, designs, results and plots are saved in the folder *Reproduction Example.* Hence, running this script reproduces Figure 2 and Figure 3 shown in the paper.

The results presented in Section 5 (Study Example) and in the Appendix (A.1, A.5, and A.7) can be reproduced by running the corresponding R scripts, as outlined above.

## Helper scripts

Helper functions are contained in the file `0_util.R`. The file is organized into the following sections:

-   **Trial simulation:** Functions for simulating study entry times, survival outcomes, and censoring data for treatment and control groups under specified distributional assumptions. It also includes a function that generates a dataset at a given interim timepoint.

-   **Test statistics:** A wrapper function around `survdiff()` that extracts the normally distributed version of the log-rank test statistic from the *survival* package. Additionally, a function for computing the inverse normal test statistic is provided. Note, however, that in the paper’s Methods section, we use the cumulative test statistic for testing.

-   **Survival parameters:** Functions for translating between different parameterizations of survival models—such as event probabilities, hazards, and median survival times—under the assumption of an exponential distribution.

-   **Study design:** A wrapper function that constructs a study design, including its boundary set, and defines unblinding boundaries for the hybrid designs.

-   **Event estimation:** Functions for estimating the expected number of events at a given time, based on assumptions about survival and censoring. These functions are used to compute the boundaries of the hybrid designs, as described in Sections 2.2.4–2.2.6 of the paper.

For details, we refer to the comments in the corresponding code file.

## Working with intermediate results

The data files are large (several GB) and therefore not included with the code. However, all result files generated by `3_evaluation.R` have been uploaded to the corresponding results folder, as outlined above. Consequently, running `4_plots.R` will reproduce all plots shown in the paper without the need to re-run the full data simulation.

## Session Info

The example results presented in this paper that were run on a local computer using the following R packages and their corresponding versions:

```{r}
sessionInfo()
```
```
