# Supplementary material for: Blinded‐Into‐Unblinded Interim Analyses for Clinical Trials With Time‐to‐Event Endpoints
Source: Biom J. 2026 Jul 24;68(4):e70151. doi: 10.1002/bimj.70151 (PMC13397411; doi:10.1002/bimj.70151)
Supplement: Supplementary file 2 — Supporting File 2: bimj70151‐sup‐0002‐Datacode.zip. [file BIMJ-68-e70151-s001.zip › R_Code_Submission_wo_data/Simulation Study/Plots/Performance/Study 1_Pocock/Figure3.pdf]

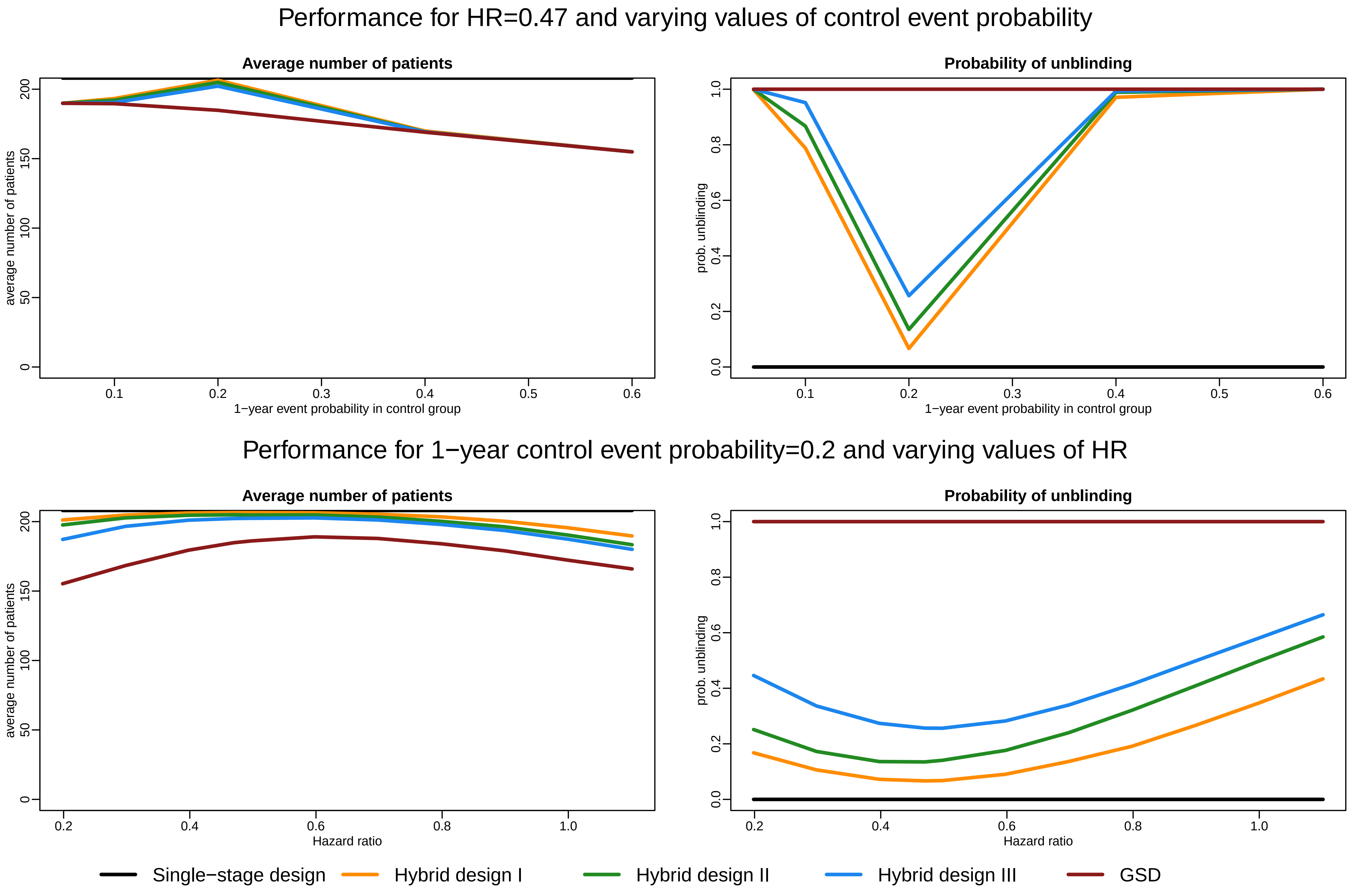
Performance for HR=0.47 and varying values of control event probability
Average number of patients
average number of patients
0
50
100
150
200
0.1
0.2
0.3
0.4
0.5
0.6
1−year event probability in control group
Probability of unblinding
prob. unblinding
0.0
0.2
0.4
0.6
0.8
1.0
0.1
0.2
0.3
0.4
0.5
0.6
1−year event probability in control group
Performance for 1−year control event probability=0.2 and varying values of HR
Average number of patients
average number of patients
0
50
100
150
200
0.2
0.4
0.6
0.8
1.0
Hazard ratio
Probability of unblinding
prob. unblinding
0.0
0.2
0.4
0.6
0.8
1.0
0.2
0.4
0.6
0.8
1.0
Hazard ratio
Single−stage design
Hybrid design I
Hybrid design II
Hybrid design III
GSD
