# Supplementary material for: Blinded‐Into‐Unblinded Interim Analyses for Clinical Trials With Time‐to‐Event Endpoints
Source: Biom J. 2026 Jul 24;68(4):e70151. doi: 10.1002/bimj.70151 (PMC13397411; doi:10.1002/bimj.70151)
Supplement: Supplementary file 2 — Supporting File 2: bimj70151‐sup‐0002‐Datacode.zip. [file BIMJ-68-e70151-s001.zip › R_Code_Submission_wo_data/Simulation Study/Plots/Type-1 Error Rate/n=360/Figure9-1.pdf]

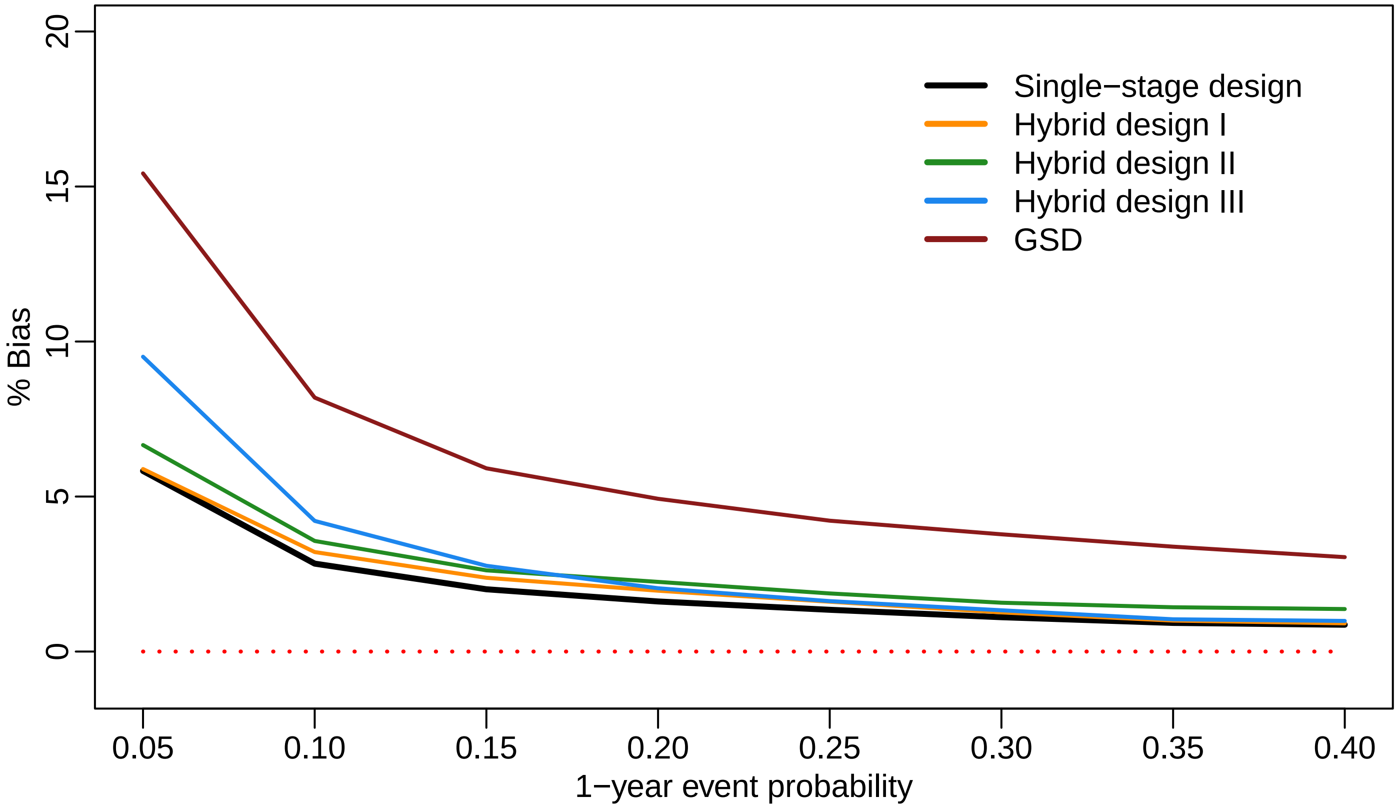

Single−stage design
Hybrid design I
Hybrid design II
Hybrid design III
GSD
% Bias
0
5
10
15
20
0.05
0.10
0.15
0.20
0.25
0.30
0.35
0.40
1−year event probability
